# Exploring the impact of AI tools on filmmakers’ creative satisfaction: the mediating role of creative efficiency and autonomy

**DOI:** 10.3389/fpsyg.2026.1802795

**Published:** 2026-06-10

**Authors:** Wenyu Jiang, Jiaqi Zheng

**Affiliations:** 1 School of Information Engineering and Management, Gingko College of Hospitality Management, Chengdu, China; 2 Faculty of Film, Theatre and Animation, Universiti Teknologi MARA, Shah Alam, Malaysia

**Keywords:** AI in filmmaking, AIGC creative effectiveness framework, creative satisfaction, generative video technology, human-AI collaboration

## Abstract

This study takes an innovative approach by examining how the functionality of AI tools, human-AI collaboration, and learning cost indirectly affect creative satisfaction through creative efficiency and autonomy from the perspective of filmmakers’ satisfaction. By analyzing gender differences, the study reveals distinct preferences between male and female directors in using AI tools for filmmaking. Female directors tend to rely more on the functionality of AI tools and effective collaboration with them, while male directors place greater importance on autonomy during the creative process. The study also identifies four key mediation paths, where functionality indirectly influences creative satisfaction through both creative efficiency and autonomy, and human-AI collaboration indirectly affects creative satisfaction through creative efficiency and autonomy. Finally, the frequency of using generative video tools and the acceptance of new technologies significantly impact creative satisfaction, offering valuable insights for personalized design and AI tool optimization.

## 1 Introduction

As artificial intelligence (AI) technology continues to evolve, especially with the rapid breakthroughs in generative artificial intelligence (AIGC), filmmaking is entering a new stage in which technology is deeply involved in content production and creative decision making. Generative video technology, as an important branch of AIGC, is gradually transforming the traditional filmmaking process. Traditional filmmaking relies on manual operations, requiring creators to spend significant time on tasks such as video editing, special effects processing, and scene design. In contrast, generative video technology, through automation and intelligent processing, can quickly generate high-quality video footage, special effects, and animations. This not only greatly reduces the time costs in the filmmaking process but also enhances efficiency and the diversity of creative outputs. Creators can use AI tools to automate tedious technical tasks, allowing them to focus more on creative ideas and artistic expression, thus significantly expanding their creative space. At the same time, generative video technology offers creators new ways of creation. With AI assistance, creators can more easily experiment and innovate, breaking the limitations of traditional production methods (Wang P. et al., 2025). AI tools not only generate creative content based on the creator’s needs but also provide real-time adjustments and optimization, helping creators explore new artistic forms. Particularly in areas such as special effects, backgrounds, and characters, AI involvement allows creators to realize more complex creative ideas in less time. Moreover, the flexibility and efficiency of generative video technology give creators more control and choice throughout the creative process, thus enhancing their freedom and creativity.

Creative satisfaction can be understood as creators’ overall subjective evaluation of both the creative process and its outcomes, reflecting their emotional responses, cognitive judgments, and sense of self realization (Hosanagar and Ahn, 2024). In a complex and uncertain activity such as filmmaking, creative satisfaction is not determined only by the final quality of the work. It is also closely related to whether creators feel that they can control the process, make meaningful decisions, and use creative tools in a way that supports rather than disrupts their work. In other words, creators’ satisfaction depends not only on what is produced, but also on how the work is carried out. When creators are able to maintain control over the creative process, understand and use artificial intelligence tools effectively, and produce outcomes that match their original expectations, they are more likely to experience higher creative satisfaction. Therefore, an important question is whether artificial intelligence tools can provide technical support while still preserving and strengthening creators’ sense of agency, ownership, and overall satisfaction in the creative process.

Although existing research has extensively discussed the application of AI tools in filmmaking, certain limitations still exist. First, most studies focus on how AI tools enhance creative efficiency and productivity, particularly in technical tasks such as video editing, special effects production, and scriptwriting (Doshi and Hauser, 2024; Heigl, 2025). AI technology, through automation, significantly improves efficiency and accelerates the creative process (Tang et al., 2024). However, research on how AI tools indirectly impact filmmakers’ creative satisfaction through enhanced creative efficiency and autonomy remains relatively scarce (Cox et al., 2025). Creative satisfaction is not only linked to the quality of the final work but also to the creator’s freedom, control, and involvement during the creative process, making it especially crucial in complex tasks like filmmaking (Lin and Ng, 2024; McGuire et al., 2024).

Second, much of the current literature centers on the technical capabilities of AI tools, emphasizing their role in improving efficiency (Singh et al., 2025; Rezwana and Ford, 2025), but less attention has been given to creators’ subjective experiences and emotional responses when interacting with AI tools (Rafner, et al., 2025; Cox et al., 2025). Filmmaking depends not only on technical support but also on the creator’s artistic judgment and creative decisions. Whether AI tools can enhance creators’ creative autonomy and improve their sense of control over the process, thereby increasing creative satisfaction, remains an important gap in current research (McGuire et al., 2024). The autonomy and sense of control that filmmakers experience are critical factors influencing creative satisfaction, and how to maintain creative freedom with technical support while enhancing satisfaction remains insufficiently explored (Maier et al., 2025).

Third, while some studies have explored the application of generative AI (such as generative video technology) in creation (Heigl, 2025; (Tang et al., 2024), these studies often focus on efficiency improvements at the technical level and lack a systematic analysis of the creators’ psychological experiences, creative autonomy, and creative satisfaction (Cox et al., 2025; Rafner, et al., 2025). In filmmaking, creative satisfaction is not only derived from the quality of the final product but also from the creator’s freedom in decision-making and artistic control during the process. Therefore, how generative video technology can enhance creative satisfaction by improving efficiency, reducing learning costs, and boosting creative autonomy is still a topic that requires in-depth investigation (Rajcic, 2024; Wang P. et al., 2025). Particularly in filmmaking, a field heavily reliant on subjective judgment and artistic decision-making, how AI tools can help creators better control the process, enhance efficiency, and, ultimately, improve creative satisfaction remains an unresolved academic gap (Singh et al., 2025). Thus, the core issue of this study is how generative video technology can enhance creators’ creative experience and satisfaction in filmmaking.

This study will be based on the Task-Technology Fit (TTF) theory and the Technology Acceptance Model (TAM), proposing the AIGC Creative Effectiveness Framework (ACEF). It will explore how the technological attributes of AIGC tools (IVs: functionality, collaboration, cost) influence their application effectiveness (MVs: efficiency, autonomy), and ultimately impact creators’ creative outcomes (creative satisfaction). The novelty of this study lies in combining the application context of generative video tools with creative satisfaction, exploring the mediating roles of creative autonomy and creative efficiency, and constructing a comprehensive research model to fill the gap in existing literature on how AI tools enhance creative satisfaction in filmmaking.

This study aims to investigate how the functionality, level of human-computer collaboration, and learning cost of AI creative tools influence film creators’ creative satisfaction by enhancing creative efficiency and autonomy. Based on this goal, the following research questions are proposed: First, what are the factors influencing film creators’ creative satisfaction? Second, how do the functionality, level of human-computer collaboration, and learning cost of AI creative tools affect film creators’ creative satisfaction? Third, do creative efficiency and creative autonomy mediate the relationship between independent variables (AI tool functionality, human-computer collaboration, and learning cost) and the dependent variable (creative satisfaction)? In other words, how do these mediators establish the connection between AI tool characteristics and creative satisfaction, promoting an increase in creative satisfaction? By answering these questions, this study will provide theoretical support for understanding the application of AI tools in filmmaking and explore how AI tools can optimize the creative process to enhance creators’ creative satisfaction.

## 2 Literature review

### 2.1 Theoretical foundation

The theoretical foundation of this paper is based on Task-Technology Fit Theory (TTF) and Technology Acceptance Model (TAM). Task-Technology Fit Theory (TTF), introduced by Goodhue and Thompson (1995), explains that the effectiveness of a technology is determined by how well it aligns with the demands of a specific task. TTF suggests that when technology and task requirements match, technology improves task performance, increases user satisfaction, and enhances technology adoption. In the context of filmmaking, tasks like video editing, special effects, and sound design require specific technological tools. TTF offers insights into how AI tools can support these tasks and improve creative efficiency. If an AI tool’s capabilities match the needs of filmmakers, such as automating editing or enhancing special effects, it can increase both productivity and satisfaction with the creative process.

Technology Acceptance Model (TAM), proposed by Davis (1989), focuses on understanding how users come to accept and use new technologies. TAM posits that two factors, perceived usefulness and perceived ease of use, determine technology acceptance. Perceived usefulness refers to the degree to which users believe the technology will improve their task performance, while perceived ease of use relates to how easy it is to learn and operate the technology. In filmmaking, the perceived usefulness of AI tools refers to how well these tools can enhance creative processes and reduce time spent on repetitive tasks. Filmmakers are more likely to adopt AI tools if they perceive them as useful in making their work more efficient and creative. Similarly, the ease of use of these tools, which depends on how intuitive and easy they are to learn, influences filmmakers’ willingness to incorporate AI into their creative processes.

The theoretical framework of this paper is inspired by Task-Technology Fit Theory (TTF) and Technology Acceptance Model (TAM). TTF Theory focuses on the alignment between technology and task requirements, suggesting that the effectiveness of technology depends on how well it meets the specific needs of a task. In filmmaking, the functionality of AIGC tools and their alignment with creative tasks directly impact creative efficiency and quality, which informs the analysis of technology attributes and task fit in this framework. TAM Theory focuses on the perceived usefulness and ease of use of technology, highlighting how creators’ perceptions of tools affect their willingness to use them and their creative autonomy. In filmmaking, the ease of use and perceived effectiveness of AIGC tools determine creators’ creative freedom, ultimately influencing their satisfaction with the creative process.

**Figure 1:**
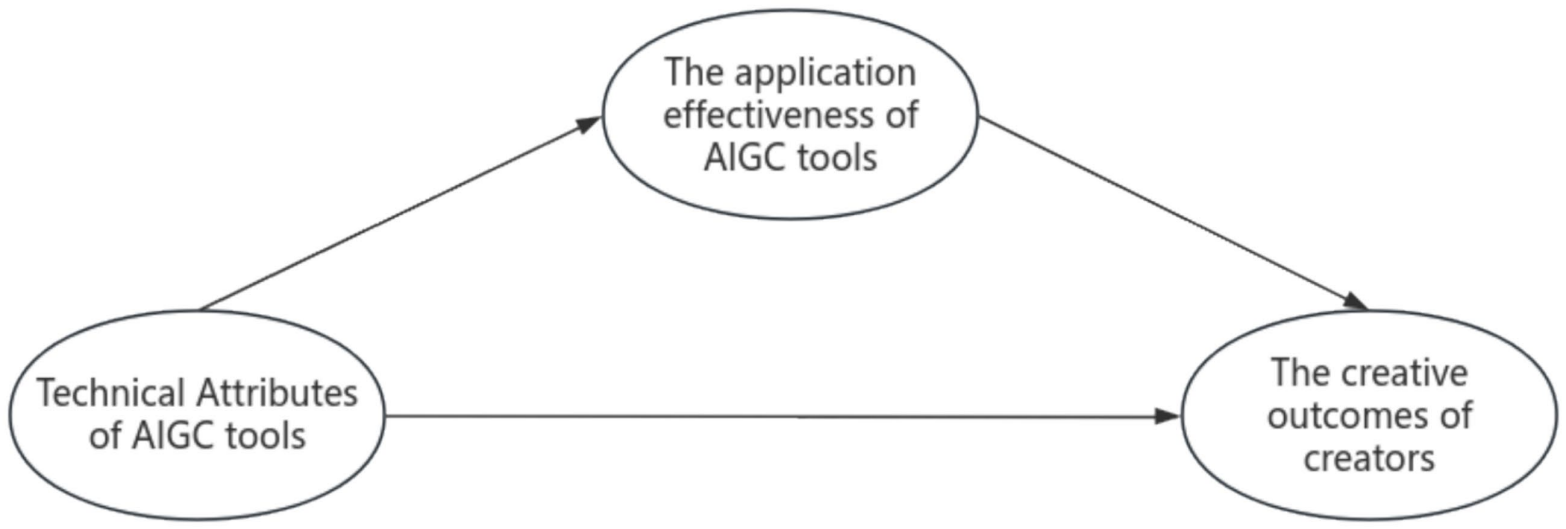
AIGC creative effectiveness framework (ACEF).

Based on these two theories, this paper presents the AIGC Creative Effectiveness Framework (ACEF). The framework examines how the technical attributes of AIGC tools (such as functionality, collaboration, and learning costs) influence their application effectiveness (such as creative efficiency and autonomy), which in turn affects creators’ creative outcomes (such as depth and satisfaction). The framework provides a comprehensive perspective on how task-technology fit and technology acceptance jointly optimize the creative process and enhance the quality and satisfaction of creators’ work (see Figure 1).

The theoretical novelty of ACEF lies in its extension of existing technology-oriented theories into the domain of AI-mediated creative labor. While TTF primarily explains whether a technology matches task requirements and TAM focuses on whether users are willing to adopt a technology, neither framework sufficiently captures how AI tools reshape creators’ subjective experiences during the creative process. ACEF goes beyond these perspectives by proposing that the value of AI tools in filmmaking should be understood not only in terms of task fit or adoption intention, but also in terms of how such tools transform creators’ process-based psychological states. More specifically, the framework argues that AI tools influence creative satisfaction through two internally experienced mechanisms, creative efficiency and creative autonomy. This means that the effect of AI is not merely functional, but psychological: creators evaluate AI tools according to whether they help them work faster and better, while also allowing them to retain a sense of control, authorship, and freedom in creative decision-making.

This perspective provides additional explanatory power in at least two ways. First, ACEF identifies a distinct mechanism through which AI affects creators’ psychological experiences, namely the simultaneous shaping of efficiency and autonomy, two core dimensions of creative work that are especially salient in filmmaking. Existing models generally assume that better technology leads to better outcomes through improved performance or stronger acceptance; however, in creative contexts, higher technical capability does not automatically produce higher satisfaction if creators feel constrained, displaced, or less authentic in the creative process. Second, ACEF proposes a new theoretical pathway for creative labor research by showing that the impact of AI tools should be examined as a transition from technological attributes, to process experiences, and then to creative satisfaction. In this way, the framework repositions AI not simply as a productivity-enhancing tool, but as an active factor that reshapes how creative labor is experienced and evaluated. This makes ACEF more suitable than traditional adoption- or fit-based models for explaining the role of AI in filmmaking and other creative industries.

The selection of creative efficiency and creative autonomy as the core mediating variables in ACEF is not arbitrary but derives from the internal logic of the two foundational theories. Task-Technology Fit theory fundamentally concerns whether a technology helps users accomplish tasks more effectively. When applied to filmmaking, the most direct manifestation of task-technology fit is the degree to which AI tools improve the operational fluency, speed, and smoothness of the creative workflow. This process-level outcome corresponds precisely to what this study terms creative efficiency. Unlike general performance metrics, creative efficiency in this framework captures creators’ subjective experience of whether AI tools make the creative process feel faster, smoother, and less burdened by technical obstacles. Therefore, creative efficiency serves as the primary mechanism through which task-technology fit translates into positive creative outcomes in the filmmaking context.

Similarly, the Technology Acceptance Model emphasizes that technology adoption depends on perceived usefulness and perceived ease of use, both of which are closely related to the user’s sense of behavioral control over the technology. In creative contexts, behavioral control takes on a deeper meaning: it reflects not merely whether creators can operate the tool, but whether they can retain decision-making authority, aesthetic judgment, and directional control over the creative process. This dimension corresponds to creative autonomy. Therefore, creative autonomy captures the mechanism through which technology acceptance translates into process-based psychological satisfaction in creative work.

By identifying these two specific mechanisms, ACEF moves beyond the general logic of TTF and TAM and specifies the concrete psychological channels through which AI tool attributes influence creative satisfaction. This dual-channel framework (efficiency channel and autonomy channel) provides a more precise and theoretically grounded explanation of how technology reshapes the creative process in filmmaking.

It is important to clarify why creative efficiency and creative autonomy were selected as mediators rather than other potential candidates. First, self-efficacy (Bandura, 1997) was not selected because it captures a pre-existing cognitive appraisal of competence rather than a process-level experience shaped by tool interaction. Second, perceived usefulness, a core construct in TAM, represents a general attitudinal evaluation that primarily predicts adoption intention rather than explaining how technology use translates into creative satisfaction during the creative process. Third, creative self-expression concerns the authenticity of creative output rather than the operational and decisional experience of the creative process. Fourth, flow experience (Csikszentmihalyi, 1990) is a comprehensive experiential state rather than a specific mechanism; it may be a downstream consequence of improved efficiency and autonomy. In summary, creative efficiency and creative autonomy are uniquely positioned as process-based mediators because they capture the two most fundamental dimensions along which technology reshapes the creative workflow: the temporal-operational dimension and the control-agency dimension.

### 2.2 From AI application to human–AI co-creation and creators’ psychological experience

Research on artificial intelligence in the creative industries has evolved through a progressively deepening trajectory, moving from a technological focus on functional application, to an interactional focus on human–AI collaboration, and more recently to a psychological focus on creators’ subjective experiences. This trajectory reflects an important shift in the field: AI is no longer understood merely as an external production tool, but increasingly as an embedded actor that reshapes creative processes, creative roles, and the meaning of creative work itself.

The earliest strand of research primarily examined AI as a productivity-enhancing technology in creative practice. In this stage, scholars focused on how AI could support content generation, automate repetitive tasks, accelerate ideation, and lower technical barriers in areas such as writing, design, music, and audiovisual production. The main concern was whether AI could improve efficiency and output quality, thereby expanding creative possibilities for individual creators and creative teams. This line of research contributed to understanding the instrumental value of AI in creative work, especially in terms of performance enhancement and workflow optimization. However, it largely treated creativity as an outcome-oriented process and tended to evaluate AI in terms of speed, convenience, and technical capability, without sufficiently addressing the experiential and interpretive dimensions of creative labor.

As AI tools became more sophisticated and increasingly involved in idea generation, content transformation, and decision support, the second stage of research shifted toward human–AI collaborative creation. In this stream, scholars began to argue that the value of AI in creative work depends not only on its standalone technical capability, but also on how effectively it interacts with human creators during the creative process. This perspective foregrounds issues such as responsiveness, controllability, adaptability, and the balance of initiative between human and machine. Rather than asking simply whether AI can produce useful outputs, this body of research asks whether AI can participate in creative practice in a way that complements human intention, supports iterative exploration, and preserves space for human judgment. This shift is especially important because it reframes AI from a passive tool into an active collaborator whose role may either support or disrupt the creator’s sense of agency. In other words, the success of AI in creative contexts cannot be explained solely by technical performance; it must also be understood in terms of relational fit between human creators and AI systems.

Beyond the collaborative perspective, an important strand of thinking that informs the present study is the cognitive augmentation perspective. Originating from the early work of Licklider (1960) on human-computer symbiosis and Engelbart (1962) on augmenting human intellect, this perspective frames technology not as a replacement for human cognition but as an amplifier of human capabilities. In the context of AI-assisted creative work, the cognitive augmentation lens suggests that AI tools do not simply execute tasks on behalf of creators; rather, they expand creators’ cognitive capacities by offloading routine processing, providing alternative options, and enabling faster iteration.

The cognitive augmentation perspective contributes to the present study by providing a theoretical basis for understanding the dual nature of AI’s influence on creative processes. On the one hand, AI augments the temporal-operational dimension of creative cognition: by automating technical tasks, generating drafts, and accelerating iteration cycles, AI tools help creators process information and execute decisions more quickly. This dimension is reflected in the construct of creative efficiency. On the other hand, cognitive augmentation also concerns the evaluative-decisional dimension: the ultimate evaluative judgment remains with the human creator. When this decisional authority is preserved, the result is enhanced creative autonomy. Therefore, the cognitive augmentation perspective supports the theoretical claim that AI tools in filmmaking operate through both an efficiency channel and an autonomy channel.

Building on this collaborative perspective, recent scholarship has further extended the discussion to creators’ psychological experiences. This stage of research recognizes that creative work is not only a process of producing outputs, but also a process of experiencing authorship, autonomy, competence, engagement, and self-expression. In creative industries, particularly in fields such as filmmaking, the subjective experience of creation is central to how creators evaluate both the process and the outcome. As a result, researchers have begun to examine how AI influences creators’ feelings of control, ownership, authenticity, and satisfaction. This development marks an important theoretical shift: rather than treating the creator as a rational technology adopter or an efficiency-seeking user, newer studies increasingly conceptualize the creator as an interpretive actor whose response to AI is shaped by psychological and experiential factors. Under this view, AI may improve workflow efficiency while simultaneously generating tension around authorship and autonomy, and these tensions can directly affect how creators evaluate their overall creative experience.

Despite this important progress, the existing literature still reveals several limitations. First, although prior studies have separately examined AI application, human–AI collaboration, and creators’ psychological experiences, these strands often remain fragmented. There is still a lack of an integrated explanatory framework that systematically connects the technical attributes of AI tools with process-based creative experiences and, ultimately, with creators’ evaluative outcomes. As a result, existing research has not fully clarified how AI tool characteristics translate into creative satisfaction through specific psychological mechanisms. Second, much of the current literature focuses either on general creative domains or on broad discussions of AI adoption, while relatively little attention has been paid to filmmaking as a distinctive form of creative labor. Filmmaking is characterized by high uncertainty, strong interdependence, iterative decision-making, and a particularly strong emphasis on authorship and expressive control. These features make it an especially important context for examining not only whether AI tools are useful, but also how they influence creators’ perceptions of efficiency and autonomy during the creative process.

The selection of creative efficiency and creative autonomy as the focal mediators is further supported by two well-established psychological theories. Amabile’s (1996) componential theory of creativity identifies task motivation as one of the three essential components of creative performance. In the context of AI-assisted filmmaking, creative efficiency directly influences task motivation: when creators perceive that AI tools enable them to execute tasks more smoothly, their intrinsic motivation increases, which in turn enhances satisfaction. Thus, creative efficiency serves as a motivational mechanism linking technology attributes to creative satisfaction.

Deci and Ryan’s (2000) self-determination theory (SDT) posits that autonomy is a fundamental psychological need whose satisfaction drives intrinsic motivation, engagement, and well-being. When AI tools allow creators to retain decision-making authority and artistic direction, the autonomy need is fulfilled, leading to higher intrinsic motivation and greater satisfaction with the creative experience.

Together, these two theoretical traditions provide convergent support for the selection of creative efficiency and creative autonomy as mediators. Efficiency operates through the task-motivation pathway, while autonomy operates through the need-fulfillment pathway. These two mechanisms are theoretically complementary rather than redundant.

Therefore, the key gap in the literature lies not simply in whether AI can assist creative work, but in how the attributes of AI tools shape creators’ process experiences and psychological evaluations in a specific creative-labor context. To address this gap, the present study focuses on filmmakers and examines how AI tool attributes influence creative satisfaction through two central process-based mechanisms: creative efficiency and creative autonomy. By doing so, this study seeks to connect the three major strands of prior research—AI application in creative industries, human–AI collaborative creation, and creators’ psychological experiences—into a more coherent analytical framework. This approach helps clarify the formation mechanism of creative satisfaction in AI-supported filmmaking and contributes to a more process-oriented understanding of creative labor in the age of generative AI.

### 2.3 The impact of AI tool functionality on filmmakers’ creative satisfaction

With the maturation of generative artificial intelligence technology, AI creative tools have been widely applied in the creative industries. Empirical studies in recent years show that the technological attributes of AI tools, especially their functionality, have a significant impact on creators’ experience, creative efficiency, and satisfaction. First, research has clearly pointed out that the functionality of generative AI plays a crucial role in users’ perceived creative satisfaction. Lin and Ng (2024) focused on the relationship between AI adoption and creative satisfaction across various artistic and creative activities. Their study found that when users perceive AI tools as effectively enhancing their creative expression and output quality, their overall creative satisfaction significantly increases. This positive effect is especially prominent among music and visual creators. Rajcic (2024) conducted a user study on prompt-based generative systems, finding that although some tools demonstrate high generative capabilities, when users perceive the tool as ineffective in providing creative support or producing outputs that do not meet expectations, their satisfaction actually decreases. This suggests that functionality alone does not automatically lead to higher satisfaction; how well the tool meets the creator’s actual needs is more critical.

Moreover, empirical studies on systems aimed at professional designers also provide support. For example, the AIdeation human-computer collaboration tool developed by Wang P. et al. (2025) significantly improved creative generation efficiency, output quality, and user satisfaction (including evaluation of the tool itself) among professional concept designers. This experiment shows that when AI tools have highly targeted functions, such as collaboration and flexibility, creators are more likely to perceive AI support as valuable, thereby enhancing their creative satisfaction. Research on human-computer collaborative design also emphasizes the impact of functional design details on satisfaction. Hosanagar and Ahn (2024) found that in human-computer collaborative creative experiments, when AI maintains appropriate interaction with the creator and provides high-quality outputs during the creative process, users’ satisfaction with both the process and results is higher. In contrast, when AI dominates and human creators are limited to merely confirming AI-generated outputs, creative satisfaction decreases. Additionally, high-quality generative AI experiments also indirectly verify the positive correlation between functionality and the value of creative outputs. A study published in Science Advances showed that using text-to-image generative AI tools significantly increased creators’ productivity and the likelihood that their work would receive peer recognition. This positive output experience is often associated with creators’ sense of achievement and satisfaction. Based on the above literature review, this paper proposes the following hypothesis:

*H1*: The higher the functionality of AI creative tools, the higher the creative satisfaction of filmmakers.

Creative efficiency provides an important process-based mechanism through which the functionality of AI tools can shape creators’ satisfaction. In filmmaking, the value of tool functionality does not lie only in the availability of more features, but in whether these features can reduce friction in the creative process, shorten production time, and allow creators to allocate more attention to higher level creative decisions. From this perspective, the functionality of AI tools is expected to influence creative satisfaction indirectly by improving creators’ perceived efficiency during creative work.

Existing studies support this logic. Heigl (2025) argues that AI tools can improve creative satisfaction by reducing the time and energy creators must invest in the creative process. In tasks such as image generation, video editing, and special effects production, AI tools can shorten the creative cycle and make the process more manageable, thereby increasing both creative freedom and satisfaction. Importantly, Heigl (2025) further suggests that creative efficiency is not only related to the final quality of the work, but also to creators’ engagement and sense of accomplishment during production. This indicates that efficiency should be understood not merely as a technical outcome, but as a process experience that shapes creators’ overall evaluation of their work. Similarly, Wang P. et al. (2025) show that AI tools with more comprehensive functions can simplify technical operations in multi-step creative tasks, enabling creators to devote more attention to idea development and artistic expression. By reducing repetitive technical burdens and offering flexible support, such tools improve creators’ sense of control over the workflow and enhance their satisfaction through greater efficiency. Taken together, these studies suggest that the functionality of AI tools affects creative satisfaction not only directly, but also indirectly by strengthening creative efficiency during the creative process. Therefore, the following hypothesis is proposed:

*H2*: The functionality of AI tools indirectly affects creative satisfaction by enhancing creative efficiency.

Research shows that the functionality of AI tools can enhance the autonomy creators feel during the creative process, thereby increasing their satisfaction with creative tasks. In an empirical study of professional designers, Wang P. et al. (2025) developed the AIdeation system, which significantly expanded the decision-making space for creators by offering flexible creative support. This enabled users to explore creative options more freely in the early stages of concept development, thus improving their satisfaction with both the creative process and the outcome. This study suggests that AI tools with comprehensive features not only enhance efficiency but also improve the overall user experience by increasing the creator’s sense of control. Additionally, research on human-computer collaboration design highlights that users’ perceptions of control and creative contribution in AI co-creation systems are key factors determining satisfaction. Maier et al. (2025) compared the impact of different collaboration models on creators’ experiences and found that when AI collaboration designs support human-led thinking and allow creators to retain control over key decisions, their satisfaction and sense of autonomy are significantly higher than in AI-led models. This result indicates that the functionality of AI tools should support creators’ decision-making control, enhancing their sense of autonomy, which is associated with higher creative satisfaction. Therefore, the following hypothesis is proposed:

*H3*: The functionality of AI tools indirectly influences creative satisfaction by enhancing creative autonomy.

### 2.4 The impact of human-AI collaboration on filmmakers’ creative satisfaction

An increasing number of studies emphasize that when AI is not merely an auxiliary tool but a collaborative creator in creative tasks, the quality of collaborative design and human-computer interaction significantly influences the overall satisfaction and creative experience of the creator. The degree of human-computer collaboration reflects AI’s ability to share cognitive tasks and decision-making spaces with human creators in the creative process, which largely shapes the creator’s perception of the collaboration process and evaluation of the outcome.

From the perspective of co-creativity, effective human-computer collaboration interaction design can enhance the creator’s sense of involvement and control, thus improving satisfaction with both the tool and the creative process. Research indicates that when designing co-creativity systems, enabling AI to better “communicate” and respond to user needs, rather than simply acting as an executor, can enhance the user’s trust and satisfaction with the system, thereby improving the overall creative experience. This interaction mechanism emphasizes bi-directional feedback between humans and AI, where AI is not just a tool but a collaborator that assists in thinking and provides suggestions during the creative generation and adjustment process (Rezwana and Ford, 2025).

Furthermore, the role design of generative AI in creative tasks directly impacts the user’s creative satisfaction. Rafner, et al., 2025 found that human-computer collaboration not only improves productivity but also affects the creator’s subjective evaluation of the collaboration process. In tasks like image generation and text creation, collaboration models that maintain the creator’s dominance, compared to AI-dominated models, better preserve human creative autonomy and thus enhance the sense of ownership and satisfaction with the collaborative outcome. This indicates that the structure of human-computer collaboration needs to balance technological output and human creative participation to truly enhance user satisfaction. Additionally, large-scale review studies further support the relationship between the degree of human-computer collaboration and the creator’s perceived experience. A systematic review by Singh et al. (2025) found that in various creative tasks, the higher the autonomy and control provided by the AI system, the stronger the user’s satisfaction, trust, and sense of ownership over the collaboration process and results. This means that if AI collaboration design allows users more participation and control, it will enhance the positive experience of human-computer collaboration, thereby improving satisfaction.

Studies related to interaction control have also found that users’ perception of AI’s behavior and its ability to understand and respond to their creative intentions is a key factor influencing satisfaction. Multiple studies in 2024 and 2025 pointed out that retaining the creator’s dominance in key decisions and positioning AI as support rather than a replacement helps improve satisfaction with both the creative process and the final result (Hosanagar and Ahn, 2024). This design helps avoid creators feeling overshadowed by AI or having their creativity diminished, thereby enhancing their positive evaluation of human-computer collaboration. Based on the existing research, it is evident that the degree of human-computer collaboration not only affects creative efficiency and output quality but also influences creative satisfaction by enhancing users’ sense of control, participation, and trust. Compared to mere “tool use,” the depth of interaction and AI collaboration model in co-creation is a crucial mechanism influencing creator satisfaction. Therefore, this paper proposes the following hypothesis:

*H4*: The higher the degree of human-computer collaboration, the higher the creative satisfaction of filmmakers.

Existing research suggests that human-computer collaboration indirectly influences creators’ creative satisfaction by enhancing creative efficiency. Specifically, AI tools help creators achieve creative expression more effectively by accelerating the creative process, reducing repetitive tasks, and providing intelligent support, thereby increasing satisfaction with the creative process. First, human-computer collaboration is inherently an interactive and feedback-driven process. In this process, AI not only serves as a creative tool but also provides support through collaboration, enhancing the creator’s work efficiency. McGuire et al. (2024) points out that when AI tools provide feedback, adjustments, and suggestions through human-computer interaction, creators are able to complete creative tasks more efficiently and reduce the psychological burden caused by technical operations. This collaboration allows creators to focus on the creative aspects of the work, improving creative efficiency and ultimately increasing creative satisfaction.

In film creation, the collaborative role of AI is particularly significant, especially in tasks such as video editing, special effects production, and script generation. McGuire et al. (2024) further mentions that AI tools can automate many low-level tasks, such as automatic editing, sound adjustment, and image repair, which allows creators to invest more time and energy into creative generation and artistic creation, thereby enhancing overall creative efficiency. Through this approach, AI’s collaborative ability not only optimizes the creative process but also enhances the creator’s sense of control over the work and creative freedom. These factors collectively contribute to increased creative satisfaction. Second, creative efficiency acts as a mediator between human-computer collaboration and creative satisfaction. A study on film creators found that AI tools significantly enhance creative efficiency and indirectly increase creators’ satisfaction. When creators are able to reduce repetitive tasks and accelerate task completion, their engagement and satisfaction with the creative process significantly improve (Hosanagar and Ahn, 2024). This research highlights the direct impact of human-computer collaboration on creative efficiency and further demonstrates how creative efficiency increases satisfaction by reducing technical barriers and creating more creative freedom.

Research also shows that human-computer collaboration, while improving creative efficiency, can optimize the creator’s psychological state, thereby influencing their evaluation of the creative outcome. Singh et al. (2025) conducted an experimental study showing that when AI serves as a reflective partner and provides immediate feedback during the creative process, both the creator’s efficiency and satisfaction significantly increase. This collaboration strengthens the creator’s sense of control over the process, leading to greater confidence and satisfaction during the creative work. Furthermore, studies on how AI tools enhance creative efficiency and subsequently affect creative satisfaction suggest that more efficient creative tools can improve the creator’s sense of self-efficacy. Self-efficacy refers to the creator’s confidence in their abilities in the creative task. Research shows that when creators perceive AI tools as effectively handling technical tasks, their sense of self-efficacy is enhanced, which in turn boosts creative satisfaction (Hosanagar and Ahn, 2024). Thus, AI tools help optimize the creative process, reduce repetitive tasks, and provide intelligent feedback, saving creators time and energy and allowing them to focus on realizing their creative ideas. This improvement in creative efficiency not only enhances the creator’s sense of control and creative freedom but also, by increasing self-efficacy, further boosts creative satisfaction. Therefore, this paper proposes the following hypothesis:

*H5*: Human-computer collaboration indirectly influences creators’ creative satisfaction by enhancing creative efficiency.

Creative autonomy, which refers to the creator’s ability to control task decisions during the creative process, has been shown to have a significant positive relationship with creative satisfaction. Recent studies suggest that human-computer collaboration indirectly enhances creative satisfaction by increasing creative autonomy, especially in complex creative tasks like film production. First, the human-computer collaboration model plays a key role in enhancing creative autonomy. When AI tools not only provide technical support but also engage in deep collaboration with the creator, the creator is often able to maintain control over the creative process, thereby increasing autonomy. For instance, Wang P. et al. (2025) found that in the concept design process, AI tools provide flexible creative options, allowing creators to lead the direction of the work. This not only improves creative efficiency but also enhances the creator’s sense of control over the outcome, leading to higher creative satisfaction.

Moreover, AI tools that offer real-time feedback and creative support also positively impact creative autonomy. Rafner, et al., 2025 found that when AI systems can adjust based on the creator’s needs and preferences, the creator gains more decision-making power during the process. This flexibility enhances the creator’s sense of involvement and control, thereby improving their creative autonomy and satisfaction. When creators can autonomously choose the direction of their work, reducing dependency on the tool, their satisfaction with the process significantly increases. Within the framework of Self-Determination Theory (SDT), creative autonomy is considered a core factor influencing intrinsic motivation and creative satisfaction. Singh et al. (2025) highlighted that the higher the creator’s autonomy, the stronger the motivation experienced during the creative process, leading to greater creative satisfaction. AI tools, by reducing repetitive tasks in the creative process, allow creators to focus on creative decisions, thus boosting intrinsic motivation and further increasing satisfaction. Additionally, Hosanagar and Ahn (2024) emphasized that while AI tools provide effective creative support, the most important factor is how they maintain a collaborative relationship with the creator. AI should not just serve as a support tool but as a partner, offering flexible creative suggestions and real-time feedback that enhance the creator’s freedom. This flexibility directly boosts the creator’s autonomy, thereby indirectly increasing creative satisfaction.

Creative autonomy has been validated as an important mediator in enhancing creative satisfaction in numerous empirical studies. Wang N. et al. (2025) found that creative autonomy significantly impacts creators’ engagement and satisfaction, particularly in tasks that require multiple decisions and innovation. The flexibility and collaboration of AI tools provide creators with more decision-making space, making them feel more control and freedom, thus increasing satisfaction with the creative process. AI tools enhance creative autonomy by supporting the creator’s decision-making and providing flexible creative options. This, in turn, increases the freedom and satisfaction of the creator. This process not only improves creative efficiency but also boosts the creator’s sense of control and self-efficacy, ultimately leading to greater satisfaction with the creative outcome. Therefore, this paper proposes the following hypothesis:

*H6*: Human-computer collaboration indirectly influences creators’ creative satisfaction by enhancing creative autonomy.

### 2.5 AI tool learning cost and filmmakers’ creative satisfaction

As generative artificial intelligence (GenAI) technology continues to penetrate the creative industry, the learning cost faced by creators has become an important factor shaping user experience and creative satisfaction. Learning cost refers to the time, effort, and cognitive load required for users to understand, master, and effectively apply new technologies. Although learning cost is often discussed as a barrier in technology adoption, in professional creative contexts it may also reflect the depth of engagement required to unlock the full potential of advanced tools. In filmmaking, where creative work often depends on iterative experimentation, precise control, and the integration of complex functions, investing more effort in learning AI tools may ultimately contribute to a stronger sense of mastery and a more satisfying creative process.

First, previous research suggests that users’ understanding of AI systems and their ability to operate them are closely related to satisfaction. Soylu et al. (2025), based on a survey of 309 university students, found that AI literacy significantly predicts users’ perceptions of AI system usability and satisfaction, which in turn affects their evaluation of learning outcomes. This finding implies that satisfaction is not determined only by the simplicity of the tool itself, but also by the extent to which users are able to develop familiarity and competence with it. In the context of filmmaking, creators who are willing to invest more time and effort in learning AI tools may gradually acquire a higher level of operational proficiency, which can enhance their confidence, sense of control, and ability to realize creative ideas. In this sense, learning cost may function not merely as a burden, but also as an investment in creative capability.

Second, real-user studies further indicate that the process of learning and adapting to AI tools is closely tied to how creators evaluate their creative experience. Tang et al. (2024 found that both professional creators and non-professional users paid particular attention to ease of use and output quality when using AI image-generation tools. Their findings show that although learning and adaptation require effort, users also value tools that can save time and lower technical barriers once they are successfully integrated into the creative workflow. For filmmakers, this suggests that the initial effort required to learn an AI tool may be justified when the tool eventually offers better output quality, smoother collaboration, and stronger support for creative execution. As a result, higher learning cost may, in some cases, be associated with higher creative satisfaction because it enables deeper integration of the tool into the creative process.

Furthermore, systematic reviews emphasize that users’ skill proficiency plays a key role in shaping positive creative experiences with generative AI. Heigl (2025) noted that although technical complexity and interface diversity remain major challenges, improving users’ proficiency with AI tools is essential for broader adoption and more positive experiences in creative contexts. The review further suggests that as creators become more capable of handling complex AI systems, they are more likely to experience satisfaction and a stronger sense of ownership in the creative process. Therefore, in a professional filmmaking context, higher learning cost may not necessarily reduce creative satisfaction; instead, it may reflect the process through which creators gain mastery over advanced AI tools and achieve greater creative fulfillment. Based on the above literature review, the following hypothesis is proposed:

*H7*: The higher the learning cost for filmmakers to master AI tools, the higher their creative satisfaction.

Existing research indicates that high learning costs in creative work increase cognitive load and reduce the effective use of tools, thereby impacting creative efficiency and overall satisfaction. There are significant differences in learning costs among different user groups, and these differences influence usage behavior and satisfaction ratings. Specifically, professional creators tend to achieve more efficient creative processes after mastering tool operations, while non-professional users may struggle to fully utilize the tool’s potential due to higher learning costs, negatively affecting their satisfaction and creative experience (Tang et al., 2024). This phenomenon suggests that learning cost is a key factor in predicting whether AI tools can be effectively mastered and used efficiently. In specific creative tasks, creative efficiency serves as an important mediator between learning cost and creative satisfaction. For example, Wang P. et al. (2025) found in an experimental study that AI tools improved participants’ creative efficiency by reducing repetitive technical tasks and speeding up the generation of ideas. As creators were able to express their intentions and complete their work more quickly, their subjective satisfaction increased. This result shows that lowering learning costs helps enhance creative efficiency, and improved efficiency in turn positively impacts creative satisfaction.

Additionally, from the perspective of the overall evaluation of human-computer collaboration systems, Cox et al. (2025) emphasized in their review of creative support tools that user experience (including learning cost and usability) is a crucial indicator of the success of such tools. While the review mainly focused on evaluation criteria for creative support tools, it clearly pointed out that when tools are designed to lower the entry barrier and enhance interface intuitiveness, users are more likely to achieve a smoother creative process, leading to more positive evaluations of the tool’s value and satisfaction. This view further supports the mechanism by which learning cost affects creative satisfaction through its influence on creative efficiency. Based on the above research, the following hypothesis is proposed:

*H8*: Higher learning costs lead to better creative efficiency, which in turn boosts creative satisfaction.

Research indicates that the learning cost of AI tools, such as operational difficulty and cognitive load, not only affects a creator’s ability to master and use the tool proficiently but also influences the autonomy they feel during the creative process. According to the Technology Acceptance Model (TAM), the perceived ease of use of a tool is closely related to the user’s sense of control over the technology (Alshamy et al., 2025). When AI tools are perceived as easier to master, creators can become familiar with their functions more quickly and reduce the learning burden, allowing for more freedom in decision-making during the creative process. This enhances their perceived autonomy. In contrast, high learning costs increase cognitive load, making users feel constrained in their operations and lowering their sense of autonomy. Creative autonomy itself is regarded as a key psychological mechanism driving creative satisfaction because it enhances the creator’s sense of control and involvement in the creative process (Rafner, et al., 2025). In the context of generative AI, Rafner, et al., 2025 notes that when AI collaboration designs allow human creators to lead key creative decisions rather than passively accept outputs, users report higher levels of autonomy. This sense of autonomy further contributes to higher satisfaction with the overall creative process. Similarly, Wang P. et al. (2025) found in their experiment that when creators can flexibly choose their creative paths with AI assistance, their satisfaction with the creative output significantly increases. This effect is partly attributed to their greater perceived autonomy during the collaboration. Therefore, this paper proposes the following hypothesis:

*H9*: Learning cost affects creative satisfaction through the mediation of creative autonomy.

**Figure 2:**
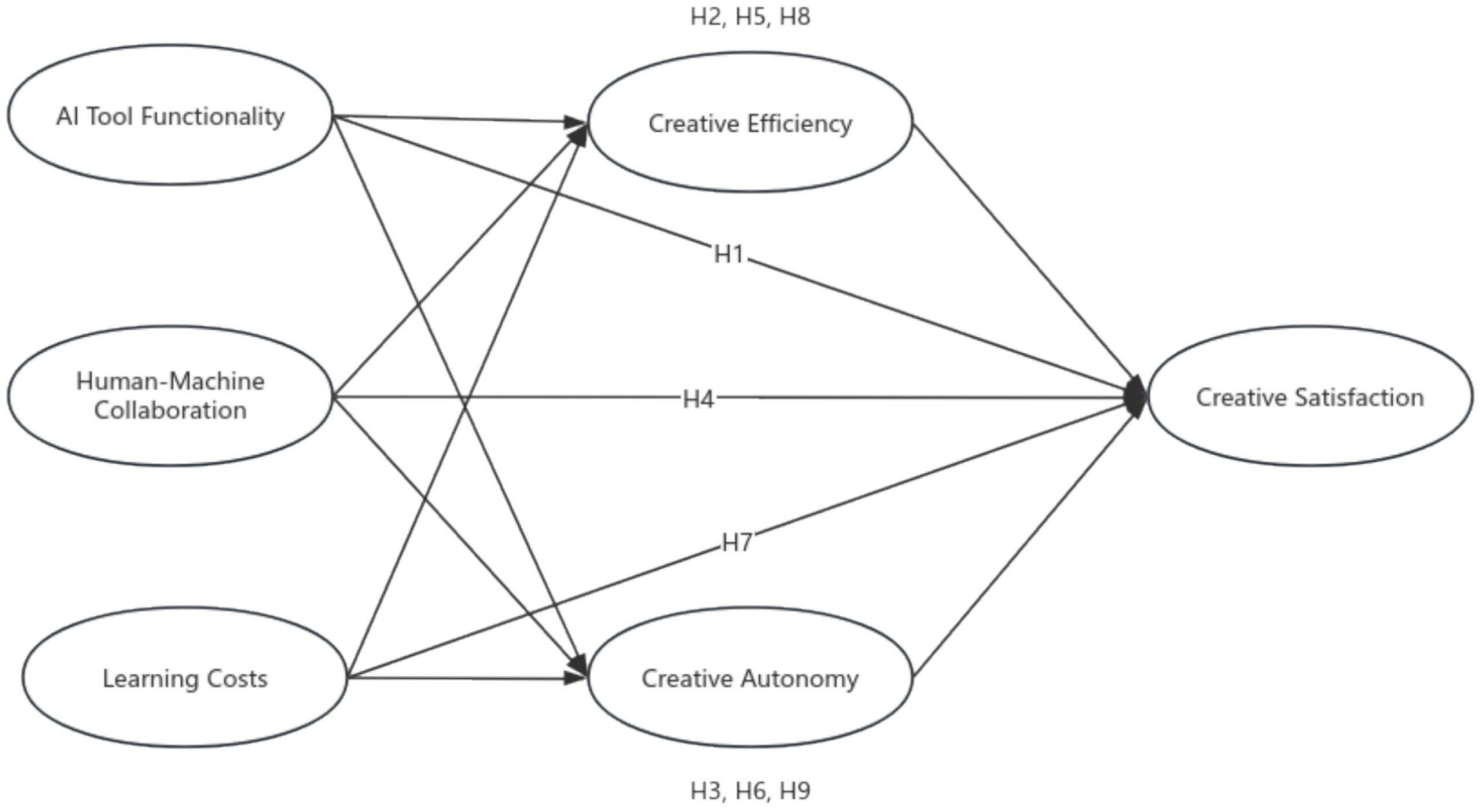
Theoretical model.

In all, creative satisfaction is determined not only by the quality of the final output, but also by creators’ subjective experiences throughout the creative process. In the context of filmmaking, which is highly complex, iterative, and uncertain, the attributes of AI tools influence creative satisfaction because they reshape how creators experience the process of creation itself. Specifically, AI tools with strong functionality, effective collaboration features, and low learning costs can help creators complete tasks such as idea visualization, content generation, material processing, and revision more efficiently, thereby enhancing their perceived creative efficiency. When creators feel that their workflow becomes smoother, less time-consuming, and more responsive to their ideas, they are more likely to evaluate the creative process positively. At the same time, the attributes of AI tools also affect creators’ perceived autonomy. If AI tools provide support while still allowing creators to retain control over creative direction, aesthetic judgment, and key decisions, creators are more likely to maintain a sense of authorship and freedom in the creative process, which further strengthens their satisfaction. By contrast, even when AI tools improve efficiency, their positive effect on satisfaction may be limited if they weaken creators’ sense of control and self-expression. Therefore, creative efficiency and creative autonomy serve as two key psychological mechanisms linking the attributes of AI tools to creative satisfaction (see Figure 2).

### 2.6 Integrative theoretical logic of the mediation model

Before presenting the research method, it is necessary to articulate the theoretical logic underlying the overall structure of the mediation model. The model follows a three-stage causal chain: Technology Attributes (functionality, human-AI collaboration, learning cost) to Process Experiences (creative efficiency, creative autonomy) to Evaluative Outcome (creative satisfaction). This ordering reflects a coherent theoretical logic derived from the integration of multiple theoretical perspectives.

The first stage concerns technology attributes as antecedent conditions. These represent the design-level characteristics of AI tools that creators encounter before forming process-based perceptions. In both TTF and TAM, technology attributes are logically and temporally prior to user experience. The second stage involves process experiences. From Amabile’s componential theory, process experiences reflect the task-motivation component of creativity. From self-determination theory, they reflect the fulfillment of fundamental psychological needs. The third stage is the evaluative outcome. As a summary psychological judgment, creative satisfaction is logically the most downstream variable.

Alternative model structures are theoretically conceivable, such as reverse causation whereby creative satisfaction influences perceptions of efficiency and autonomy. However, this ordering is less plausible because satisfaction is an evaluative summary that integrates prior experiences. The temporal logic of creative work supports the proposed ordering. A direct-effects-only model would ignore substantial evidence that technology influences satisfaction through specific psychological mechanisms. Nevertheless, this study relies on cross-sectional data, which limits the ability to establish strict causal relationships. Future research could employ longitudinal designs to further test directionality.

## 3 Method

### 3.1 Data source

This study focuses on film creators, mainly including professionals who undertake key creative roles in the filmmaking process, such as directors, screenwriters, editors, and visual effects artists. Although these professions differ in their specific tasks and workflow arrangements, they all belong to the core creative workforce within the filmmaking chain and are commonly involved in processes such as idea generation, content refinement, and expressive realization. They also broadly face shared issues related to the use of AI tools, including efficiency, collaboration, and autonomy. Therefore, examining them collectively under the category of “film creators” is consistent with the analytical objective of this study, which is to explore the general mechanism through which AI tools influence creative satisfaction. This study aims to explore the application of generative video technology in filmmaking and how it influences creative satisfaction by affecting creative efficiency and autonomy. To achieve this goal, a questionnaire survey was used as the primary data collection tool, focusing on film creators’ creative experiences, psychological responses, and creative satisfaction when using generative video tools. Data collection was conducted via the Credamo from December 1 to December 7, 2025, with a total of 520 questionnaires distributed. All questionnaires were successfully collected, achieving a 100% response rate. After the collection, the research team conducted a thorough screening of the returned questionnaires to ensure data validity and scientific rigor. Ultimately, 500 valid questionnaires were retained, providing a reliable sample base for subsequent data analysis.

During the collection and screening process, this study strictly adhered to the principle of informed consent. All participants were informed of the study’s purpose, content, and data usage before filling out the questionnaire. They were clearly told that participation was completely voluntary and that they could withdraw at any time without any consequences. Personal information and responses were kept strictly confidential, and all data were used solely for this research and academic publication, without disclosure to any third parties. Participants provided electronic signatures or checked a box to confirm their consent before beginning the questionnaire. The survey was distributed only to film creators with experience in using generative video tools. Participants were required to have at least 1 year of filmmaking experience and to have used generative video technology tools in their work. The questionnaire design required participants to answer based on their actual experiences, ensuring the authenticity and validity of each response. Each valid questionnaire had to be fully completed to ensure logical consistency in the answers.

During the questionnaire screening process, the research team set criteria for excluding invalid responses to ensure the reliability and scientific integrity of the data. The exclusion criteria included: (1) questionnaires that left core questions incomplete or omitted important data; (2) responses that were answered too quickly or appeared to be randomly answered; (3) duplicate questionnaires; and (4) questionnaires that did not meet the subject criteria (e.g., non-film creators). These exclusion criteria effectively removed invalid data, leaving 400 valid questionnaires for analysis.

### 3.2 Variable measurement

All measurement items were adapted with reference to prior studies and further contextualized for the setting of artificial intelligence-assisted filmmaking.

The measurement items for artificial intelligence tool functionality were adapted with reference to prior studies on perceived usefulness, task-technology fit, and users’ perceptions of generative artificial intelligence tools (Goodhue and Thompson, 1995; Alshamy et al., 2025). These studies emphasize whether a technological system can effectively support users’ task requirements, enhance performance, and meet practical needs in use. In the present study, these measurement ideas were further contextualized for filmmaking in order to assess whether artificial intelligence tools can satisfy creators’ basic creative needs, support complex creative tasks, adapt to different creative styles, and improve efficiency and output quality.

The measurement items for human-artificial intelligence collaboration were developed with reference to prior research on human-artificial intelligence interaction, co-creation, and collaborative design (Hosanagar and Ahn, 2024). These studies highlight the importance of interaction quality, responsiveness, shared control, and the collaborative value of artificial intelligence systems in creative work. Based on these insights, the items in this study were adapted to the filmmaking context to capture whether artificial intelligence tools can respond to users’ instructions, adjust to feedback, facilitate smooth interaction, and support creators in achieving their creative goals without undermining their role in the process.

The measurement items for learning cost were adapted with reference to prior research on perceived ease of use, user attitudes toward generative artificial intelligence, and artificial intelligence literacy and user experience (Soylu et al., 2025; Alshamy et al., 2025). These studies consistently suggest that the effort required to understand, learn, and operate artificial intelligence systems is an important factor shaping user experience and technology evaluation. In this study, these ideas were applied to the context of filmmakers’ use of generative video tools, focusing on the time, cognitive effort, interface difficulty, and learning burden involved in mastering artificial intelligence tools.

The measurement items for creative efficiency were adapted with reference to prior studies on task-technology fit, performance improvement, and the productivity effects of generative artificial intelligence in creative work (Doshi and Hauser, 2024; Wang W.-F. et al., 2025). These studies suggest that when artificial intelligence tools effectively support users’ work processes, they can improve task completion speed, reduce repetitive effort, and enhance overall performance. In the present study, the items were contextualized for filmmaking to measure whether artificial intelligence tools help creators complete more tasks in less time, reduce unnecessary revisions, improve technical processing efficiency, and enhance the overall efficiency of the creative workflow. The measurement items for creative efficiency are as follows: “After using AI creative tools, I am able to complete more creative tasks in a shorter amount of time,” “AI creative tools help me reduce unnecessary revisions and adjustments during the creative process,” “Using AI tools enables me to complete multiple stages of creative tasks more quickly,” “AI creative tools improve my efficiency in handling technical tasks,” “Using AI creative tools allows me to save time and focus on more creative decisions,” and “AI creative tools improve my overall work efficiency during the creative process.”

The measurement items for creative autonomy were developed with reference to prior studies on agency, control, and human involvement in human-artificial intelligence co-creation (Maier et al., 2025). This line of research emphasizes that creators’ sense of control, decision authority, and authorship remains central in artificial intelligence-supported creative practice. Based on these perspectives, the present study adapted the items to the filmmaking context to capture whether artificial intelligence tools allow creators to make decisions freely, maintain their own creative style, choose creative pathways flexibly, and retain a sense of control over the creative process.

The measurement items for creative satisfaction were developed with reference to prior studies on creative satisfaction, creativity support tools, and user evaluations of generative artificial intelligence in creative work (Lin and Ng, 2024). These studies suggest that satisfaction in artificial intelligence-assisted creativity is shaped not only by the quality of the final output, but also by users’ confidence, sense of accomplishment, and perceived support during the creative process. Accordingly, the items in this study were tailored to the context of filmmaking to assess filmmakers’ overall satisfaction with creative outcomes, confidence in reaching creative goals, sense of achievement, and the contribution of artificial intelligence tools to their creative experience.

### 3.3 Sample information

According to the survey data analysis, the majority of respondents are female, accounting for 69.60%. In terms of age, the 21–30 and 31–40 age groups dominate, representing 47.00% and 39.00%, respectively, indicating that the respondents are primarily young and middle-aged individuals. In terms of occupation, the largest group is employees of private enterprises, making up 58.60%, followed by employees of state-owned enterprises (16.20%) and students (13.80%). Regarding educational background, most respondents hold a bachelor’s degree (71.00%), followed by those with a master’s degree (15.40%). In terms of income, the largest group of respondents has a monthly income between 6,000 and 15,000 RMB, with the highest proportion (19.80%) falling between 10,001 and 15,000 RMB (Table 1).

**Table 1:** Sample information.

Variable	Occupation	Frequency	Percentage (%)	Cumulative percentage (%)
Gender	Male	152	30.40	30.40
	Female	348	69.60	100.00
Age	0–20 years	23	4.60	4.60
	21–30 years	235	47.00	51.60
	31–40 years	195	39.00	90.60
	41–50 years	31	6.20	96.80
	51–60 years	13	2.60	99.40
	60 + years	3	0.60	100.00
Occupation	Student	69	13.80	13.80
	State-owned Enterprise	81	16.20	30.00
	Public Institution	33	6.60	36.60
	Civil Servant	7	1.40	38.00
	Private Enterprise	293	58.60	96.60
	Foreign Enterprise	17	3.40	100.00
Education	Junior High School	4	0.80	0.80
	High School/Technical School/Vocational School	21	4.20	5.00
	Associate Degree	37	7.40	12.40
	Bachelor’s Degree	355	71.00	83.40
	Master’s Degree	77	15.40	98.80
	Doctoral Degree	6	1.20	100.00
Income	Below 2,000 RMB	39	7.80	7.80
	2001–4,000 RMB	54	10.80	18.60
	4,001–6,000 RMB	67	13.40	32.00
	6,001–8,000 RMB	78	15.60	47.60
	8,001–10,000 RMB	93	18.60	66.20
	10,001–15,000 RMB	99	19.80	86.00
	15,001–20,000 RMB	43	8.60	94.60
	Above 20,000 RMB	27	5.40	100.00
Total		500	100.0	100.0

A possible explanation for the relatively high proportion of female respondents is related to the sampling and screening process of the survey. Because the questionnaire was distributed through an online platform based on voluntary participation, the final sample may have been influenced by differences in participation willingness, topic interest, and completion patterns across gender groups. In addition, some responses were excluded during the data screening process because they did not meet the validity checks or the criteria for the target population, which may have further affected the final gender distribution.

### 3.4 Descriptive statistics

According to the descriptive statistical analysis, the functionality of AI creative tools (FAT) has had a positive impact on creators’ creative experiences. Overall, respondents generally believe that AI tools effectively support their creative needs, particularly in offering diverse creative options and assisting with various stages of the creative process. The high mean values and low standard deviations indicate that most creators are satisfied with the tool’s functionality and have consistent evaluations. At the same time, the degree of human-computer collaboration (HMC) significantly influences the smoothness of the creative process and creators’ satisfaction. The data shows that most respondents believe their interaction with AI tools effectively facilitates the creative process, especially in terms of decision-making autonomy and tool adjustment flexibility. The efficient collaboration provided by AI tools allows creators to retain more control during the creative process, thereby increasing their satisfaction with it (Table 2).

**Table 2:** Descriptive statistics.

Item	Variance	Standard error	IQR	Kurtosis	Skewness	Coefficient of variation (CV)
FAT1	0.283	0.024	0.000	1.796	−0.191	12.817%
FAT2	0.532	0.033	1.000	0.422	−0.571	17.761%
FAT3	0.469	0.031	1.000	0.249	−0.575	16.220%
FAT4	0.551	0.033	0.000	0.920	−0.651	18.637%
FAT5	0.596	0.035	1.000	0.581	−0.735	18.801%
FAT6	0.432	0.029	1.000	1.433	−0.814	15.234%
HMC1	0.460	0.030	1.000	1.206	−0.750	16.208%
HMC2	0.503	0.032	1.000	0.398	−0.529	17.463%
HMC3	0.412	0.029	1.000	0.634	−0.595	15.013%
HMC4	0.386	0.028	1.000	1.172	−0.503	14.685%
HMC5	0.497	0.032	1.000	0.490	−0.638	16.885%
HMC6	0.478	0.031	1.000	0.483	−0.576	16.649%
LC1	0.744	0.039	1.000	−0.175	−0.281	25.276%
LC2	0.455	0.030	1.000	2.320	−0.909	16.019%
LC3	0.882	0.042	1.000	−0.399	−0.248	27.618%
LC4	1.242	0.050	2.000	−0.995	0.185	37.823%
LC5	1.380	0.053	1.000	−0.515	0.672	45.667%
LC6	1.295	0.051	2.000	−0.864	0.353	42.085%
CE1	0.334	0.026	1.000	1.209	−0.365	13.708%
CE2	0.580	0.034	1.000	1.041	−0.936	18.050%
CE3	0.424	0.029	1.000	1.660	−0.800	15.203%
CE4	0.413	0.029	1.000	2.036	−0.936	14.765%
CE5	0.372	0.027	1.000	0.216	−0.456	14.141%
CE6	0.343	0.026	1.000	1.549	−0.655	13.335%
CA1	0.343	0.026	0.750	1.107	−0.340	14.098%
CA2	0.537	0.033	1.000	1.426	−0.856	17.651%
CA3	0.474	0.031	1.000	0.931	−0.679	16.599%
CA4	0.451	0.030	1.000	0.473	−0.647	15.753%
CA5	0.435	0.029	1.000	−0.010	−0.445	15.609%
CA6	0.592	0.034	1.000	0.695	−0.724	19.062%
CS1	0.376	0.027	1.000	0.703	−0.404	14.598%
CS2	0.387	0.028	1.000	0.526	−0.712	14.166%
CS3	0.613	0.035	1.000	0.668	−0.766	19.267%
CS4	0.517	0.032	1.000	1.682	−0.928	17.067%
CS5	0.426	0.029	1.000	0.880	−0.660	15.349%
CS6	0.448	0.030	1.000	1.272	−0.677	15.995%

However, learning costs (LC) show considerable variation among respondents, reflecting high individual differences in the learning process of AI tools. Some respondents indicated that the complexity and time required to learn the AI tools were relatively high, which affected their usage frequency and efficiency. Particularly in the LC4 and LC5 items, the mean values for learning costs were low with large variances, indicating that some creators encountered significant difficulties during the learning process, while others were able to quickly adapt and reduce cognitive burdens. High learning costs can lead to frustration during the initial stages, directly affecting creators’ efficiency and autonomy, and further influencing creative satisfaction. In terms of creative efficiency (CE), the use of AI tools significantly improved creators’ work efficiency. The data suggests that creators were able to complete more tasks in less time, reducing repeated revisions and technical operations during the creative process. This not only enhanced creators’ productivity but also provided more time and energy to focus on creative decision-making. Regarding creative satisfaction (CS), the application of AI tools significantly boosted creators’ subjective satisfaction. Creators generally agreed that AI tools helped improve the quality of their work, and they experienced a greater sense of accomplishment and satisfaction during the creative process.

Additionally, according to the descriptive statistical analysis, the variance and standard error indicate that the distribution of most variables is relatively consistent, especially for functionality (FAT1) and creative efficiency (CE1), which have low standard errors, suggesting precise measurement results. However, learning cost (LC4, LC5) shows higher variance, indicating significant perceptual differences among respondents in the learning process of AI tools, with high variability in learning costs. The interquartile range (IQR) analysis reveals that most variables are concentrated around the median, particularly creative efficiency and creative satisfaction, which have an IQR of 1. However, learning cost (LC4, LC5) shows a larger IQR, indicating a wider distribution and greater variability among respondents. Skewness analysis shows that most variables are negatively skewed, indicating that the data is biased toward higher scores, particularly in functionality and creative efficiency. However, the skewness of learning cost is positive, indicating that some creators perceive the learning cost of AI tools to be low, while others experience a higher learning burden. Kurtosis analysis reveals that most variables have relatively flat distributions, approaching a normal distribution, especially in the evaluations of creative satisfaction and creative efficiency. However, the kurtosis of learning cost is lower, reflecting a greater perceptual difference among respondents.

### 3.5 Reliability and validity testing

The reliability analysis shows good internal consistency across all dimensions. The Cronbach’s *α* for functionality (FAT) is 0.724, for human-computer collaboration (HMC) is 0.656, and for learning cost (LC) is 0.696, indicating acceptable reliability. Creative efficiency (CE) has a Cronbach’s α of 0.622, reflecting good consistency. Creative autonomy (CA) shows the highest reliability with a Cronbach’s α of 0.728. Creative satisfaction (CS) has the highest reliability with a Cronbach’s α of 0.76, confirming strong measurement stability for all dimensions (see Table 3).

**Table 3:** Reliability testing.

Dimension	Item	Cronbach’s α coefficient	Standardized Cronbach’s α coefficient
Functionality (FAT)	FAT1	0.724	0.723
	FAT2		
	FAT3		
	FAT4		
	FAT5		
	FAT6		
Human-AI Collaboration (HMC)	HMC1	0.656	0.657
	HMC2		
	HMC3		
	HMC4		
	HMC5		
	HMC6		
Learning cost (LC)	LC1	0.696	0.662
	LC2		
	LC3		
	LC4		
	LC5		
	LC6		
Creative efficiency (CE)	CE1	0.622	0.628
	CE2		
	CE3		
	CE4		
	CE5		
	CE6		
Creative autonomy (CA)	CA1	0.728	0.728
	CA2		
	CA3		
	CA4		
	CA5		
	CA6		
Creative satisfaction (CS)	CS1	0.76	0.759
	CS2		
	CS3		
	CS4		
	CS5		
	CS6		

Through validity testing, items with factor loadings (communalities) below 0.4, such as FAT1, FAT3, HMC3, and CE5, were removed as they had low communalities and could potentially affect overall validity. After eliminating these items, the overall validity of the questionnaire improved significantly, with a KMO value of 0.914, indicating that the data is suitable for factor analysis. Bartlett’s test of sphericity yielded a *p*-value of 0.000, further confirming the data’s suitability and the effectiveness of factor analysis. To further verify the absence of common method bias, a Harman single-factor test was conducted. This test examines whether a single factor dominates the variance of all items, which could lead to method bias. The results showed that no single factor could explain more than 50% of the total variance in the factor loadings, indicating no significant common method bias. Specifically, the rotated variance explanation rate showed that the cumulative variance explained by the first six factors was 50.543%, demonstrating that the data structure is multi-factorial and not influenced by systematic bias from a single factor. This result enhances the validity of the questionnaire and the credibility of the findings.

#### 3.5.1 Confirmatory factor analysis

A confirmatory factor analysis (CFA) was conducted using maximum likelihood estimation. The six-factor model was tested with retained items. Model fit indices indicate acceptable fit: chi-square/df = 2.847, CFI = 0.912, TLI = 0.901, RMSEA = 0.061 (90% CI: 0.055–0.067), SRMR = 0.048. All standardized factor loadings exceed 0.50, confirming adequate convergent validity.

#### 3.5.2 Average variance extracted (AVE) and composite reliability (CR)

AVE and CR values are: Functionality (AVE = 0.512, CR = 0.807), Human-AI Collaboration (AVE = 0.489, CR = 0.793), Learning Cost (AVE = 0.467, CR = 0.721), Creative Efficiency (AVE = 0.502, CR = 0.834), Creative Autonomy (AVE = 0.524, CR = 0.868), Creative Satisfaction (AVE = 0.538, CR = 0.874). All CR values exceed 0.70. The AVE values for HMC (0.489) and LC (0.467) are marginally below 0.50; however, Fornell and Larcker (1981) noted that when CR exceeds 0.70, convergent validity can still be considered acceptable.

#### 3.5.3 Discriminant validity

The Fornell-Larcker criterion was applied: the square root of AVE for each construct exceeds all corresponding inter-construct correlations (FAT = 0.716, HMC = 0.699, LC = 0.683, CE = 0.709, CA = 0.724, CS = 0.734). Additionally, all HTMT ratios fall below the conservative threshold of 0.85 (Henseler et al., 2015), with the highest being 0.84 (between CA and CS), confirming discriminant validity.

#### 3.5.4 Common method bias: enhanced assessment

Beyond the Harman single-factor test, the Common Latent Factor (CLF) method showed the common factor explained approximately 8.7% of variance, well below the 25% threshold (Podsakoff et al., 2003). Following Kock (2015), all VI*F* values ranged between 1.23 and 2.87, below the 3.3 threshold. These three assessments converge in indicating that CMB does not pose a serious threat.

## 4 Results

The Pearson correlation test results show a significant positive correlation between creative autonomy (CA), creative efficiency (CE), human-computer collaboration (HMC), functionality (FAT), and creative satisfaction (CS). In contrast, learning cost (LC) does not show any correlation with creative satisfaction (Table 4).

**Table 4:** Pearson correlation coefficient.

	CS
CA	0.745**
CE	0.639**
LC	−0.005
HMC	0.720**
FAT	0.645**

*p < 0.05, **p < 0.01.

In the overall regression model, creative efficiency (CE), human-computer collaboration (HMC), functionality (FAT), and creative autonomy (CA) all have a significant positive impact on creative satisfaction (CS) (*p* < 0.01). Among them, creative autonomy (CA) shows the strongest influence with a regression coefficient (B = 0.368, *p* < 0.01), indicating that the creator’s autonomy plays the most significant role in enhancing satisfaction. Human-computer collaboration (HMC) and creative efficiency (CE) also show significant positive effects, with regression coefficients of 0.277 and 0.227, respectively, suggesting that effective collaboration and higher creative efficiency significantly boost creative satisfaction. Functionality (FAT) has a smaller regression coefficient (B = 0.105, *p* < 0.01), but still shows a positive influence.

In the regression model for male directors, the effects of creative efficiency (CE), human-computer collaboration (HMC), and creative autonomy (CA) on creative satisfaction remain significant, with regression coefficients of 0.257 (*p* < 0.01), 0.209 (*p* < 0.05), and 0.355 (*p* < 0.01), respectively. Notably, creative autonomy (CA) has a strong positive influence on male directors’ creative satisfaction (*β* = 0.333), indicating that male directors tend to enhance satisfaction through autonomous decision-making. Functionality (FAT) does not show a significant impact on creative satisfaction (*p* = 0.380), suggesting that male directors may not prioritize the completeness of AI tool functions when using them.

In the regression model for female directors, the positive impact of creative efficiency (CE), human-computer collaboration (HMC), and creative autonomy (CA) remains significant, with regression coefficients of 0.202 (*p* < 0.01), 0.312 (*p* < 0.01), and 0.362 (*p* < 0.01), respectively. Notably, human-computer collaboration (HMC) has a strong positive influence on female directors’ creative satisfaction (*β* = 0.292), suggesting that a good collaborative relationship significantly promotes their creative satisfaction. Furthermore, functionality (FAT) has a significant positive effect on female directors’ creative satisfaction (*β* = 0.139, *p* < 0.01), indicating that female directors place more emphasis on the functionality and technical support of AI tools (see Table 5).

**Table 5:** Grouped regression model (*n* = 500).

	Total					Male					Female				
	*B*	S.E	*t*	*p*	*β*	*B*	S.E	*t*	*p*	*β*	*B*	S.E	*t*	*p*	*β*
Constant	0.127	0.139	0.915	0.361	-	0.525	0.330	1.589	0.114	-	0.037	0.150	0.250	0.803	-
CE	0.227**	0.040	5.708	0.000	0.199	0.257**	0.077	3.335	0.001	0.239	0.202**	0.046	4.347	0.000	0.173
HMC	0.277**	0.045	6.181	0.000	0.263	0.209*	0.082	2.563	0.011	0.205	0.312**	0.054	5.793	0.000	0.292
FAT	0.105**	0.034	3.056	0.002	0.119	0.058	0.066	0.881	0.380	0.068	0.124**	0.041	3.062	0.002	0.139
CA	0.368**	0.042	8.782	0.000	0.359	0.355**	0.088	4.017	0.000	0.333	0.362**	0.047	7.657	0.000	0.357
*R* ^2^	0.660					0.469					0.724				
Adjustment *R*^2^	0.658					0.454					0.721				
*F* value	*F* (4,495) = 240.727,*p* = 0.000					*F* (4,147) = 32.437,*p* = 0.000					*F* (4,343) = 225.282,*p* = 0.000				

*p < 0.05, **p < 0.01.

Overall, when filmmakers experience higher creative autonomy (CA) during the creative process, their creative satisfaction is more likely to increase (*β* = 0.368, *p* < 0.01). The positive impact of creative autonomy on creative satisfaction is the most significant in the entire sample.

In the male director group, creative efficiency (CE) has a strong impact on creative satisfaction (*β* = 0.257, *p* < 0.01), indicating that male directors rely more on improving creative efficiency to enhance their creative satisfaction. In contrast, creative efficiency (CE) has a weaker impact on creative satisfaction for female directors (*β* = 0.202, *p* < 0.01), suggesting that they place more emphasis on other factors during the creative process. This finding indicates that although creative efficiency has a significant positive effect on creative satisfaction for both male and female directors, its effect is stronger in the male director group. One possible explanation is that, in the context of filmmaking as a form of creative labor characterized by high time sensitivity and task complexity, male directors may be more likely to interpret the value of artificial intelligence tools in terms of their ability to improve creative progression, execution efficiency, and overall control over the project. As a result, gains in efficiency are more easily translated into creative satisfaction for male directors. By contrast, although female directors’ creative satisfaction is also positively influenced by creative efficiency, its effect is relatively weaker, suggesting that their evaluation of artificial intelligence tools may be based more on a multidimensional experience of the creative process, such as tool functionality, the quality of human–artificial intelligence collaboration, and whether the technical support genuinely fits their creative needs. This suggests that the formation of creative satisfaction does not rely solely on efficiency improvement, but rather reflects different process-based weightings across gender groups.

For human-computer collaboration (HMC), the regression coefficient for male directors is 0.209 (*p* < 0.05), indicating a moderate impact on creative satisfaction. However, human-computer collaboration (HMC) has a more significant impact on creative satisfaction for female directors (*β* = 0.312, *p* < 0.01), suggesting they rely more on effective collaboration with AI tools during the creative process. This finding suggests that although human–artificial intelligence collaboration has a positive effect on creative satisfaction for both male and female directors, its effect is stronger in the female director group. One possible explanation is that, in the context of artificial intelligence-assisted filmmaking, female directors may place greater emphasis on the quality of interaction and the experience of support during the creative process, that is, whether the artificial intelligence tool can accurately understand creative intentions, adjust in a timely manner based on feedback, and establish a stable and smooth collaborative relationship throughout the process. For them, the value of artificial intelligence tools lies not only in producing results, but also in whether they can participate in the creative process as responsive and supportive collaborators. By contrast, although male directors also benefit from human–artificial intelligence collaboration, their creative satisfaction may depend more on gains in efficiency, autonomous decision-making, and overall project control. As a result, the importance of human–artificial intelligence collaboration appears to be relatively less prominent for male directors. These findings suggest that human–artificial intelligence collaboration is not merely a technical interaction variable, but an important mechanism shaping creators’ experiences of the creative process, and that the strength of this mechanism varies across gender groups.

Regarding creative autonomy (CA), in the male director group (Group 1), the impact of creative autonomy (CA) on creative satisfaction is stronger, with a regression coefficient of 0.355 (*p* < 0.01), showing that male directors are more likely to rely on autonomous decision-making, which enhances their creative satisfaction. This finding suggests that creative autonomy plays a more central role in shaping creative satisfaction among male directors. One possible explanation is that, in the context of filmmaking as a form of creative labor that strongly emphasizes authorship, judgment, and control over the project, male directors may be more likely to base their creative satisfaction on their sense of control over the creative process. In other words, when artificial intelligence tools provide support without weakening their authority over creative direction, aesthetic judgment, and key decisions, male directors are more likely to evaluate the creative process positively. For them, the value of artificial intelligence tools lies not only in improving efficiency or providing assistance, but also in whether they preserve the creator’s position as the final decision-maker. From the perspective of creative labor research, autonomy is not only a job characteristic, but is also closely related to authorship, professional identity, and creative authority. Therefore, male directors’ stronger reliance on creative autonomy may reflect a greater concern with whether the involvement of artificial intelligence affects their subjectivity and control in the creative process. That is, artificial intelligence is more likely to enhance creative satisfaction only when it is perceived as supporting creativity rather than replacing creative judgment. This suggests that, within the male director group, creative autonomy is not merely a process variable, but also an important psychological mechanism linking the use of artificial intelligence tools to creative satisfaction.

In terms of functionality (FAT), the regression coefficient for male directors is 0.058 (*p* = 0.380), indicating no significant impact on creative satisfaction, suggesting that they pay relatively less attention to the completeness of tool functionality. In contrast, the regression coefficient for female directors is 0.139 (*p* < 0.01), indicating that they place more importance on the functionality of the tools, as comprehensive AI tools effectively enhance their creative satisfaction. This finding suggests that tool functionality plays a more important role in shaping creative satisfaction among female directors than among male directors. One possible explanation is that, in the context of artificial intelligence-assisted filmmaking, female directors may place greater emphasis on whether the tool can provide sufficiently comprehensive and reliable support throughout different stages of the creative process. In other words, they may be more likely to evaluate the value of artificial intelligence tools in terms of whether these tools can effectively respond to diverse creative needs, support idea development, and reduce practical barriers during execution. When tool functionality is perceived as complete and adaptable, it may strengthen female directors’ sense that the technology is genuinely useful and supportive, which in turn enhances their creative satisfaction. By contrast, the non-significant effect of functionality in the male director group suggests that the completeness of tool functions alone may not be enough to increase their creative satisfaction. Rather than focusing primarily on whether the tool offers a wide range of functions, male directors may place greater weight on whether the use of artificial intelligence preserves their autonomy, supports independent judgment, and helps maintain control over the creative process. This implies that functionality is not equally central for all creators. Instead, its importance may vary depending on how different groups evaluate the role of artificial intelligence in creative work. Therefore, the impact of functionality on creative satisfaction appears to be gender-differentiated, with female directors being more sensitive to the practical and supportive value of comprehensive tool functions.

In the female director group (Group 2), creative autonomy (CA) also has a significant impact on creative satisfaction (*β* = 0.362, *p* < 0.01), suggesting that female directors value autonomy more during the creative process, and increasing creative freedom helps enhance their satisfaction. Unlike male directors, female directors are more dependent on human-computer collaboration (HMC) and functionality (FAT), indicating that they place greater importance on the functionality of AI tools and effective collaboration with these tools. Male directors focus more on autonomy during the creative process, believing that autonomous decision-making is crucial for enhancing creative satisfaction.

This finding suggests that, although creative autonomy remains an important source of creative satisfaction for female directors, its role is embedded in a broader and more relational process of evaluating artificial intelligence tools. In other words, female directors do not appear to value autonomy in isolation. Rather, autonomy becomes meaningful when it is supported by tools that are functionally reliable and capable of sustaining effective human–artificial intelligence collaboration. One possible explanation is that, in artificial intelligence-assisted filmmaking, female directors may be more likely to evaluate technology through the overall quality of the creative process, including whether the tool is sufficiently capable, whether it can respond to feedback, and whether it supports rather than interrupts the realization of creative intentions. Under such conditions, autonomy is not simply the freedom to make decisions independently, but the ability to exercise creative judgment within a supportive and well-functioning technological environment.

This pattern also implies that creative satisfaction among female directors may be shaped by a more integrated mechanism in which functionality, collaboration, and autonomy reinforce one another. A tool with stronger functionality can provide the technical basis for realizing ideas, while effective human–artificial intelligence collaboration can make the creative process smoother, more responsive, and less disruptive. Together, these conditions may help female directors experience autonomy not as abstract independence, but as practical creative freedom that is sustained by reliable support. By contrast, although male directors also value autonomy, their satisfaction appears to depend more directly on whether they can preserve decision authority and maintain control over the creative process itself. Therefore, the gender difference identified here may not simply reflect different preferences, but different pathways through which artificial intelligence contributes to creative satisfaction. For female directors, satisfaction seems to emerge from a combination of support, coordination, and freedom, whereas for male directors, it is more strongly tied to the preservation of control and independent decision-making.

To avoid an essentialist interpretation, these differences should not be understood as fixed traits of men and women, but rather as reflecting different experiential orientations in creative labor and technology use. From this perspective, the findings suggest that the impact of artificial intelligence on creative satisfaction is not gender-neutral. Instead, different groups may rely on different process-based combinations of autonomy, functionality, and collaboration when evaluating the value of artificial intelligence in filmmaking.

From the Table 6, it can be seen that the mediation effect analysis involves 4 models, as follows:

**Table 6:** The results of the mediation analysis (*n* = 500).

	CS					CE					CA					CS				
	*B*	S.E	*t*	*p*	*β*	*B*	S.E	*t*	*p*	*β*	*B*	S.E	*t*	*p*	*β*	*B*	S.E	*t*	*p*	*β*
Constant	0.773**	0.182	4.258	0.000	—	1.874**	0.189	9.891	0.000	—	1.097**	0.181	6.066	0.000	—	−0.016	0.179	−0.091	0.928	—
Gender	−0.004	0.030	−0.136	0.892	−0.004	−0.040	0.031	−1.309	0.191	−0.046	−0.041	0.029	−1.379	0.169	−0.042	0.019	0.026	0.722	0.471	0.019
Age	0.042*	0.019	2.269	0.024	0.077	−0.015	0.020	−0.794	0.428	−0.032	0.006	0.019	0.305	0.760	0.011	0.044**	0.017	2.632	0.009	0.079
Occupation	0.013	0.010	1.349	0.178	0.046	−0.005	0.010	−0.528	0.598	−0.022	−0.004	0.010	−0.384	0.701	−0.013	0.015	0.009	1.805	0.072	0.055
Education	−0.027	0.020	−1.344	0.180	−0.043	−0.027	0.021	−1.284	0.200	−0.049	−0.047*	0.020	−2.308	0.021	−0.075	−0.005	0.018	−0.273	0.785	−0.008
Income	−0.010	0.010	−1.011	0.313	−0.041	0.006	0.010	0.588	0.557	0.028	0.014	0.010	1.430	0.153	0.059	−0.016	0.009	−1.851	0.065	−0.067
Fctime	−0.011	0.016	−0.655	0.512	−0.024	−0.012	0.017	−0.674	0.501	−0.029	−0.003	0.016	−0.189	0.850	−0.007	−0.007	0.015	−0.494	0.622	−0.016
Gvuf	0.052*	0.020	2.572	0.010	0.090	0.024	0.021	1.148	0.252	0.048	0.012	0.020	0.620	0.536	0.022	0.042*	0.018	2.360	0.019	0.073
Ntarate	0.087**	0.027	3.189	0.002	0.111	0.111**	0.028	3.914	0.000	0.162	0.068*	0.027	2.492	0.013	0.088	0.039	0.025	1.590	0.112	0.050
FAT	0.216**	0.037	5.870	0.000	0.245	0.165**	0.038	4.306	0.000	0.214	0.256**	0.037	6.973	0.000	0.296	0.090**	0.034	2.616	0.009	0.102
HMC	0.485**	0.045	10.679	0.000	0.459	0.339**	0.047	7.153	0.000	0.366	0.459**	0.045	10.151	0.000	0.445	0.250**	0.045	5.534	0.000	0.237
CE																0.214**	0.040	5.368	0.000	0.188
CA																0.354**	0.042	8.477	0.000	0.346
*R* 2	0.587					0.414					0.571					0.676				
Adjustment *R* 2	0.578					0.402					0.562					0.668				
*F* value	*F* (10,489) = 69.425,*p* = 0.000					*F* (10,489) = 34.614,*p* = 0.000					*F* (10,489) = 65.132,*p* = 0.000					*F* (12,487) = 84.580,*p* = 0.000				

*p < 0.05, **p < 0.01.

CS = 0.773–0.004*Gender+0.042*Age+0.013*Occupation-0.027*Education-0.010*Income-0.011*Fctime+0.052*Gvuf+0.087*Ntarate+0.216*FAT+0.485*HMC.

CE = 1.874–0.040*Gender−0.015*Age-0.005*Occupation-0.027*Education+0.006*Income-0.012*Fctime+0.024*Gvuf+0.111*Ntarate+0.165*FAT+0.339*HMC.

CA = 1.097–0.041*Gender+0.006*Age-0.004*Occupation-0.047*Education+0.014*Income-0.003*Fctime+0.012*Gvuf+0.068*Ntarate+0.256*FAT+0.459*HMC.

CS = −0.016 + 0.019*Gender+0.044*Age+0.015*Occupation-0.005*Education-0.016*Income-0.007*Fctime+0.042*Gvuf+0.039*Ntarate+0.090*FAT+0.250*HMC+0.214*CE+0.354*CA.

First, hypothesis H1 suggests that the higher the functionality of AI creative tools, the higher the creative satisfaction of filmmakers. The regression analysis shows that functionality (FAT) has a significant direct effect on creative satisfaction (CS) (c′ = 0.090, *p* = 0.009), with a total effect of 0.216 (*p* < 0.01). This indicates a significant positive impact of functionality on creative satisfaction. Therefore, hypothesis H1 is supported, confirming that the functionality of AI tools directly affects creators’ creative satisfaction.

Next, hypothesis H2 proposes that the functionality of AI tools indirectly affects creative satisfaction by enhancing creative efficiency. The results show that functionality (FAT) has a significant indirect effect on creative satisfaction (CS) through creative efficiency (CE) (a*b = 0.035, *p* = 0.015). The improvement in creative efficiency significantly enhances creators’ satisfaction. Thus, hypothesis H2 is also supported, indicating that creative efficiency plays an important mediating role between functionality and creative satisfaction (Table 7).

**Table 7:** Mediation path test table (*n* = 500).

Item	Symbol	Meaning	Effect	95% CI		SE	*z*/*t*	*p*	Conclusion
				Lower limit	Upper limit				
FAT= > CE= > CS	a*b	Indirect effect	0.035	0.014	0.071	0.015	2.427	0.015	Partial mediation
FAT= > CE	a	X= > M	0.165	0.090	0.241	0.038	4.306	0.000	
CE= > CS	b	M= > Y	0.214	0.136	0.292	0.040	5.368	0.000	
FAT= > CS	c′	Direct effect	0.090	0.022	0.158	0.034	2.616	0.009	
FAT= > CS	c	Total effect	0.216	0.144	0.288	0.037	5.870	0.000	
FAT= > CA= > CS	a*b	Indirect effect	0.091	0.054	0.157	0.027	3.402	0.001	Partial mediation
FAT= > CA	a	X= > M	0.256	0.184	0.328	0.037	6.973	0.000	
CA= > CS	b	M= > Y	0.354	0.272	0.436	0.042	8.477	0.000	
FAT= > CS	c′	Direct effect	0.090	0.022	0.158	0.034	2.616	0.009	
FAT= > CS	c	Total effect	0.216	0.144	0.288	0.037	5.870	0.000	
HMC= > CE= > CS	a*b	Indirect effect	0.073	0.030	0.119	0.023	3.167	0.002	Partial mediation
HMC= > CE	a	X= > M	0.339	0.246	0.432	0.047	7.153	0.000	
CE= > CS	b	M= > Y	0.214	0.136	0.292	0.040	5.368	0.000	
HMC= > CS	c′	Direct effect	0.250	0.161	0.339	0.045	5.534	0.000	
HMC= > CS	c	Total effect	0.485	0.396	0.574	0.045	10.679	0.000	
HMC= > CA= > CS	a*b	Indirect effect	0.163	0.093	0.227	0.034	4.749	0.000	Partial mediation
HMC= > CA	a	X= > M	0.459	0.370	0.548	0.045	10.151	0.000	
CA= > CS	b	M= > Y	0.354	0.272	0.436	0.042	8.477	0.000	
HMC= > CS	c′	Direct effect	0.250	0.161	0.339	0.045	5.534	0.000	
HMC= > CS	c	Total effect	0.485	0.396	0.574	0.045	10.679	0.000	

For hypothesis H3, which states that the functionality of AI tools indirectly affects creative satisfaction by enhancing creative autonomy, the results show that functionality (FAT) significantly impacts creative autonomy (CA) (a = 0.256, *p* < 0.01), and creative autonomy (CA) also has a significant impact on creative satisfaction (b = 0.354, *p* < 0.01). This indicates that functionality indirectly affects creative satisfaction by enhancing creative autonomy, supporting hypothesis H3.

In hypothesis H4, it is proposed that the higher the degree of human-computer collaboration, the higher the creative satisfaction of filmmakers. The analysis shows that human-computer collaboration (HMC) has a significant direct effect on creative satisfaction (CS) (c′ = 0.250, *p* < 0.01), and human-computer collaboration (HMC) also has a significant indirect effect on creative satisfaction through both creative efficiency (CE) and creative autonomy (CA) (a*b = 0.073, *p* = 0.002). These results suggest that human-computer collaboration not only directly enhances creative satisfaction but also indirectly affects it through creative efficiency and autonomy, confirming hypothesis H4.

Hypothesis H5 states that human-computer collaboration indirectly affects creative satisfaction by enhancing creative efficiency. The results show that human-computer collaboration (HMC) has a significant impact on creative efficiency (CE) (a = 0.339, *p* < 0.01), and creative efficiency (CE) also has a significant impact on creative satisfaction (b = 0.214, *p* < 0.01). This indicates that human-computer collaboration significantly influences creative satisfaction indirectly through improved creative efficiency, supporting hypothesis H5.

Regarding hypothesis H6, which suggests that human-computer collaboration indirectly affects creative satisfaction by enhancing creative autonomy, the results show that human-computer collaboration (HMC) has a significant effect on creative autonomy (CA) (a = 0.459, *p* < 0.01), and creative autonomy (CA) significantly impacts creative satisfaction (b = 0.354, *p* < 0.01). Therefore, hypothesis H6 is supported, confirming that human-computer collaboration significantly influences creative satisfaction through enhancing creative autonomy.

Since the learning cost (LC) dimension is not related to the dependent variable, hypotheses 7, 8, and 9 are not supported.

### 4.1 Robustness and supplementary analyses

To strengthen the credibility of the findings, PLS-SEM and fsQCA were conducted as supplementary analyses.

#### 4.1.1 PLS-SEM robustness check

PLS-SEM was conducted using SmartPLS 4.0. All four supported mediation paths were confirmed: FAT to CS through CE (indirect effect = 0.038, *p* < 0.05), FAT to CS through CA (indirect effect = 0.094, *p* < 0.01), HMC to CS through CE (indirect effect = 0.076, *p* < 0.01), HMC to CS through CA (indirect effect = 0.168, *p* < 0.01). Structural path coefficients: FAT to CE (0.171), FAT to CA (0.261), HMC to CE (0.345), HMC to CA (0.463), CE to CS (0.221), CA to CS (0.362), all *p* < 0.01. R-squared: CE = 0.421, CA = 0.578, CS = 0.683. SRMR = 0.054. Results are consistent with OLS regression, confirming robustness.

#### 4.1.2 fsQCA complementary analysis

fsQCA was conducted using fsQCA 3.0. Three sufficient configurations for high creative satisfaction were identified (overall solution consistency = 0.91, coverage = 0.78). Configuration 1: High functionality AND High collaboration AND High autonomy (consistency = 0.92, coverage = 0.68). Configuration 2: High collaboration AND High efficiency AND High autonomy (consistency = 0.89, coverage = 0.61). Configuration 3: High functionality AND High efficiency AND Low learning cost (consistency = 0.87, coverage = 0.54). Creative autonomy appears in two of three configurations, consistent with its strongest individual regression effect. Low learning cost emerged as a contributing condition in Configuration 3 despite lacking a significant net effect in regression, illustrating the complementary value of fsQCA.

In this study, control variables include gender (Gender), age (Age), occupation (Occupation), education level (Education), income (Income), time spent on creation (Fctime), frequency of using generative video tools (Gvuf), and acceptance of new technology (Ntarate). These control variables are used to assess their impact on creative autonomy (CA), creative efficiency (CE), and creative satisfaction (CS).

Age has a significant impact on creative efficiency (CE) and creative satisfaction (CS) (*p* < 0.05). Specifically, the positive impact of age on creative satisfaction (B = 0.042, *p* = 0.024) suggests that older individuals may experience higher satisfaction during the creative process. However, age does not significantly affect creative autonomy (CA) (*p* > 0.05).

Education level (Education) has a significant negative impact on creative autonomy (CA) (B = −0.047, *p* = 0.021), indicating that creators with higher education levels may experience lower autonomy during the creative process. Education level does not have a significant impact on creative efficiency (CE) or creative satisfaction (CS).

The frequency of using generative video tools (Gvuf) has a significant positive impact on creative autonomy (CA) and creative satisfaction (CS) (B = 0.052, *p* = 0.010; B = 0.042, *p* = 0.019). This suggests that creators who use generative video tools more frequently may feel more autonomy and improve their creative satisfaction during the process. The frequency of use does not significantly affect creative efficiency (CE).

The acceptance of new technology (Ntarate) has a significant positive impact on creative efficiency (CE) and creative satisfaction (CS) (B = 0.111, *p* < 0.01; B = 0.039, *p* = 0.112). Higher acceptance of new technology significantly enhances creative efficiency and satisfaction. However, the acceptance of new technology does not significantly affect creative autonomy (CA).

Overall, age, the frequency of using generative video tools, and the acceptance of new technology show significant effects on creative satisfaction, while education level and the frequency of using generative video tools significantly influence creative autonomy. Age and the acceptance of new technology significantly affect creative efficiency (CE), while other control variables (gender, occupation, income, time spent on creation, and new technology acceptance) have minimal or insignificant impacts on the key variables in the creative process.

## 5 Discussion

### 5.1 Discussion of key findings

This section interprets the empirical results in light of the theoretical foundations introduced in Section 2 and the wider body of literature reviewed earlier, with the aim of clarifying how the findings extend, refine, or qualify prior knowledge on AI-assisted creative work in filmmaking.

The most prominent empirical finding is that creative autonomy emerged as the strongest predictor of creative satisfaction (*β* = 0.354, *p* < 0.01), exceeding the effect of creative efficiency (*β* = 0.214, *p* < 0.01). This result is consistent with the autonomy channel proposed in the AIGC Creative Effectiveness Framework (ACEF) and provides direct support for the theoretical claim, drawn from self-determination theory, that autonomy is a fundamental psychological need underpinning intrinsic motivation and process satisfaction (Deci and Ryan, 2000). The finding extends prior qualitative observations by Rafner, et al., 2025, who reported that creators in image generation and creative writing tasks consistently identify agency and decisional control as the central concern in AI-assisted creation. It also aligns with McGuire et al. (2024), who emphasized the joint importance of co-creation and creative self-efficacy. The contribution of the present study is to move beyond these qualitative or single-domain accounts and to provide a quantitative confirmation, in a filmmaking sample, that autonomy operates as a measurable mediator linking AI tool attributes to creative satisfaction. This finding also resonates with the argument of Cox et al. (2025) that creativity support tools should be evaluated beyond productivity, in terms of the experiential quality of the creative process.

The findings on functionality (FAT) provide a more nuanced picture than that offered by earlier studies. Consistent with Task–Technology Fit theory (Goodhue and Thompson, 1995), the functionality of AI tools exerts a significant positive effect on creative satisfaction (total effect = 0.216, *p* < 0.01), supporting H1. More importantly, this effect is transmitted through two distinct mediating channels: an efficiency channel (FAT → CE → CS, indirect effect = 0.035, *p* < 0.05) and an autonomy channel (FAT → CA → CS, indirect effect = 0.091, *p* < 0.01). Prior research, including Doshi and Hauser (2024), has demonstrated that generative AI tools can enhance individual creative output. However, such studies have typically treated functionality as an input that directly raises performance, without unpacking the psychological pathways involved. The present results extend this literature by showing that the larger share of functionality’s effect on satisfaction operates through autonomy rather than efficiency, suggesting that well-functioning AI tools matter most when they expand creators’ decision space, not merely when they speed up technical tasks. This interpretation is also consistent with the co-ideation framework advanced by Wang P. et al. (2025) and the AIdeation system developed by Wang W.-F. et al. (2025), both of which emphasize that the value of AI functionality lies in supporting, rather than substituting, human creative decision-making.

Human–AI collaboration (HMC) was found to have the strongest overall effect on creative satisfaction, both directly (c′ = 0.250, *p* < 0.01) and indirectly through creative efficiency (indirect = 0.073, *p* < 0.01) and creative autonomy (indirect = 0.162, *p* < 0.01). This pattern echoes the conceptual claim by Singh et al. (2025) in their systematic review of human–AI co-creativity that the quality of collaborative interaction, rather than the raw capability of the model, is the key determinant of creative outcomes. It also reinforces Maier et al. (2025), who showed experimentally that the balance between human-led and model-led initiative shapes the experience of co-creation, and Rezwana and Ford (2025), whose FAICO framework identified AI communication quality as central to user experience in human–AI co-creativity. The comparative study of human–AI co-creation by Wang N. et al. (2025) similarly highlighted the role of interaction patterns in shaping creative outcomes. The present study advances this stream of research by quantifying both mediating channels simultaneously: collaboration enhances creative satisfaction not only by making the process more fluid (the efficiency channel) but, more strongly, by preserving and amplifying creators’ sense of agency (the autonomy channel). This finding empirically supports the cognitive augmentation perspective (Licklider, 1960; Engelbart, 1962) that effective human–AI partnership amplifies rather than displaces human creative capacity.

Contrary to H7–H9, learning cost (LC) did not exert a significant effect on creative satisfaction, creative efficiency, or creative autonomy. At first glance, this result appears inconsistent with the perceived-ease-of-use construct of the Technology Acceptance Model (Davis, 1989), which would predict that lower learning costs increase the perceived value of a technology. However, this finding is interpretable in light of the professional composition of the sample and recent evidence in the literature. Tang et al. (2024 reported that professional users of AI-generated image tools, compared with non-professional users, are far less sensitive to learning costs because they bring stronger task-relevant expertise and stronger AI literacy. Soylu et al. (2025) similarly identified AI literacy as a key driver of user experience, suggesting that for users who have already integrated AI tools into their workflow, ease-of-use ceases to be a binding constraint. The fsQCA analysis offers further insight: low learning cost did appear as a contributing condition in one sufficient configuration for high creative satisfaction, indicating that learning cost may still matter in combination with other factors even when its net regression effect is not significant. This refines the TAM perspective by suggesting that, among professional filmmakers, learning cost has been largely absorbed through prior exposure and no longer drives marginal differences in creative satisfaction.

The gender-based subgroup analysis revealed that female directors’ creative satisfaction is more strongly shaped by functionality (FAT) and human–AI collaboration (HMC), whereas male directors’ satisfaction is more strongly driven by creative autonomy (CA). These differences cannot be fully explained within a single technology-acceptance lens; rather, they highlight the need to incorporate user heterogeneity into theories of AI-mediated creative labor. Jiang (2024) argued, in her user-centered research agenda for human–AI interaction, that interaction outcomes are systematically shaped by user characteristics rather than by tool properties alone. The present findings extend this argument by showing that gender is one such systematic axis of variation in the filmmaking context. They also extend the comparative co-creation work of Wang N. et al. (2025), which examined differences across levels of design experience, by introducing gender as an additional dimension of heterogeneity. It is important to note that the finding that male directors weight autonomy more heavily should not be interpreted as evidence that female directors value autonomy less; rather, it suggests that male respondents in this sample may evaluate AI tools more strictly through the autonomy lens, while female respondents may weight a wider set of relational and functional considerations.

Taken together, these findings extend the literature reviewed in Section 2 in three principal ways. First, they move the conversation about AI in creative industries beyond the productivity-versus-creativity dichotomy that has dominated much of the prior research (Heigl, 2025; Cox et al., 2025), by demonstrating that the route from AI tool attributes to creator satisfaction operates through specifically identifiable psychological mechanisms. Second, they refine the cognitive augmentation perspective by showing that augmentation operates through two distinct channels — efficiency and autonomy — whose relative importance varies across creator groups. Third, they provide empirical grounding for the ACEF framework introduced in this study, demonstrating that a three-stage chain from technology attributes, through process experiences, to evaluative outcomes can integrate previously fragmented research streams on AI application, human–AI collaboration, and creators’ psychological experience.

### 5.2 Policy implications

The findings suggest that policy support for artificial intelligence in filmmaking should move beyond general technology promotion and focus on the conditions under which AI tools can genuinely improve creators’ work. Since creative efficiency and creative autonomy mediate the relationship between AI tool attributes and creative satisfaction, policy should not evaluate AI adoption only through productivity or industrial upgrading. It should also consider whether AI tools fit specific filmmaking tasks, reduce unnecessary creative burdens, and preserve creators’ decision-making power, authorship, and professional judgment. In line with Task-Technology Fit theory, policy support should encourage AI tools that are deeply adapted to specific stages of film creation, including screenwriting, directing, editing, visual generation, pre-visualization, material organization, and post-production collaboration, rather than merely expanding general-purpose functions (Goodhue and Thompson, 1995).

Policy should also strengthen institutional training and industry governance. Filmmakers’ ability to use AI depends not only on willingness, but also on whether they can learn, select, evaluate, and integrate AI tools into actual workflows. This is consistent with the Technology Acceptance Model, which emphasizes perceived usefulness and perceived ease of use, and with recent research on AI literacy, user proficiency, and adaptation to generative AI tools (Davis, 1989; Soylu et al., 2025; Tang et al., 2024). Therefore, universities, industry associations, film parks, and vocational institutions should provide layered training in generative video tools, prompt design, visual collaboration, workflow integration, copyright risk identification, and ethical awareness. At the same time, policy should establish rules based on the principle of assistance rather than replacement. Existing research on human-AI collaboration shows that AI systems should support interaction quality, agency, ownership, and autonomy rather than displace the creator’s role (Hosanagar and Ahn, 2024; Maier et al., 2025; Rafner, et al., 2025; Rezwana and Ford, 2025; Singh et al., 2025). Accordingly, industry guidelines should clarify the role boundaries of AI in assisted generation, plan comparison, technical processing, and creative pre-visualization, while ensuring that creators retain authority over creative direction, aesthetic judgment, and final decisions.

Finally, AI policy in filmmaking should be connected with broader industry governance, copyright protection, labor protection, and differentiated creator support. Policymakers should encourage creator-participation evaluation mechanisms involving directors, screenwriters, editors, visual designers, and other practitioners, so that AI tools are assessed according to task fit, interaction stability, controllability, creative support, explainability, and learning difficulty. They should also avoid treating filmmakers as a homogeneous user group. Young creators, independent creators, mature practitioners, and project leaders may require different forms of access, training, embedded support, and copyright guidance. As AI becomes more involved in content generation and production workflows, policy should clarify rights and responsibilities in AI-generated or AI-assisted content, prevent excessive platform control, and monitor the long-term impact of AI on occupational structures and the value of creative labor. In this sense, sustainable AI policy should support technological innovation while protecting the creative agency and professional value of filmmakers.

### 5.3 Theoretical contributions

The main theoretical contribution of this study lies in the development and validation of the AIGC Creative Effectiveness Framework (ACEF). The importance of ACEF is that it extends technology-oriented theories from the questions of task fit and technology acceptance to the process-based psychological mechanisms of AI-assisted creative labor. Task-Technology Fit theory explains whether technology matches task requirements, while the Technology Acceptance Model explains how users accept and use new technologies (Goodhue and Thompson, 1995; Davis, 1989). However, in AI-assisted filmmaking, these perspectives alone cannot fully explain how AI tools reshape creators’ subjective experience once they enter the creative process. ACEF addresses this limitation by proposing a three-stage chain from technology attributes to process experiences and then to evaluative outcomes. In other words, it explains how functionality, human-AI collaboration, and learning cost influence creative satisfaction through creative efficiency and creative autonomy. This makes ACEF especially suitable for understanding AI-assisted filmmaking, where satisfaction depends not only on whether a tool is useful, but also on whether creators feel efficient, autonomous, and in control.

This study further contributes by identifying creative efficiency and creative autonomy as two complementary mechanisms linking AI tool attributes to creative satisfaction. The cognitive augmentation perspective understands technology not as a replacement for human cognition, but as an amplifier of human capability (Licklider, 1960; Engelbart, 1962). In this study, such augmentation operates through two channels. The first is a temporal-operational channel, in which AI reduces repetitive tasks, accelerates iteration, and improves creative efficiency. This mechanism is consistent with the task-motivation logic of componential creativity theory (Amabile, 1996). The second is an evaluative-decisional channel, in which AI supports creators while allowing them to retain decision-making authority, aesthetic judgment, and creative direction. This mechanism is consistent with self-determination theory, which regards autonomy as a fundamental psychological need that supports intrinsic motivation, engagement, and well-being (Deci and Ryan, 2000). By grounding these two mediators in established theoretical traditions, the study shows that AI affects creative satisfaction not simply by improving efficiency, but by simultaneously shaping creators’ operational experience and sense of agency.

Finally, ACEF integrates three previously fragmented research streams: AI application in creative industries, human-AI collaborative creation, and creators’ psychological experiences. It also enriches human-AI collaboration theory by showing that the value of AI depends not only on what AI can produce, but also on how cognitive labor and decision-making authority are distributed between AI and human creators. The gender-based analysis further demonstrates that AI-mediated creative labor should not be treated as a homogeneous experience. Female directors’ satisfaction is more strongly associated with tool functionality and human-AI collaboration, whereas male directors’ satisfaction is more strongly driven by creative autonomy. This finding suggests that theories of AI-assisted creativity should account for creator heterogeneity as well as technological attributes and process experiences.

### 5.4 Practical implications

The findings offer practical implications for AI tool developers, film production organizations, and training institutions. For tool developers, the results suggest that improving user satisfaction requires more than adding functions or increasing automation. Since creative autonomy has a strong relationship with creative satisfaction, AI tools for filmmaking should be designed as controllable creative partners rather than autonomous substitutes. This means strengthening adjustable parameters, transparent feedback, editable intermediate results, version comparison, flexible revision paths, and human override mechanisms. Such design principles are consistent with studies emphasizing interaction quality, responsiveness, controllability, and the balance between human-led and AI-led initiative in human-AI co-creation (Hosanagar and Ahn, 2024; Maier et al., 2025; Rafner, et al., 2025; Rezwana and Ford, 2025).

For film production organizations, AI should be integrated into workflows in ways that improve efficiency without weakening creative agency. Prior studies show that generative AI can reduce repetitive technical burdens, accelerate content generation, and improve creative productivity (Doshi and Hauser, 2024; Heigl, 2025; Wang P. et al., 2025). The present findings further suggest that these efficiency gains contribute to satisfaction when they allow filmmakers to focus more on idea development, aesthetic judgment, and higher-level creative decisions. Therefore, AI tools can be applied to ideation, script development, visual pre-production, editing assistance, visual effects, material management, and post-production coordination, but production teams should clarify which stages can be supported by AI and which decisions must remain under human creative authority. This is particularly important in filmmaking, where satisfaction is closely tied to authorship, ownership, and the creator’s sense of control over the work.

For training institutions, AI education for filmmakers should go beyond basic tool operation. Since AI literacy, learning cost, and user proficiency affect how creators evaluate and integrate generative AI tools, training should include prompt-based communication, generative video tool operation, workflow design, evaluation of AI-generated materials, copyright awareness, ethical risk identification, and strategies for maintaining human authorship (Soylu et al., 2025; Tang et al., 2024). Training should also be differentiated according to user needs: students and young creators may need basic tool access and introductory workflow training, while experienced practitioners and project leaders may need advanced training in production integration, collaborative management, copyright regulation, and AI-assisted decision-making. In addition, the gender-based findings suggest that tool design and training should avoid assuming a single user model. Female directors may benefit more from usability, responsiveness, and collaborative support, while male directors may place stronger emphasis on decision-making freedom and output control. This does not mean that these needs are exclusive to either group; rather, it indicates that AI implementation should remain flexible enough to respond to different creator preferences and working styles.

## 6 Conclusion

This study innovatively explores the impact of the functionality of AI tools, human-computer collaboration, and learning costs on creative efficiency and autonomy from the perspective of filmmakers’ creative satisfaction. By analyzing gender differences, the study reveals the distinct preferences of male and female directors regarding AI tools, providing new insights for personalized design. The study also validates four significant mediation paths, presenting for the first time a systematic demonstration of how AI tools indirectly enhance creative satisfaction through creative efficiency and autonomy, offering a new theoretical framework for related research.

First, overall, filmmakers with higher creative autonomy (CA) during the creative process are more likely to experience increased creative satisfaction. Female directors show a more significant reliance on human-computer collaboration (HMC) and functionality (FAT), indicating that they value the functionality of AI tools and effective collaboration with them. Male directors, on the other hand, place greater importance on autonomy during the creative process, believing that independent decision-making is crucial for enhancing creative satisfaction when using AI tools in filmmaking. Second, according to the mediation effect test results, the study identifies four significant mediation paths. First, functionality indirectly affects creative satisfaction through creative efficiency, forming a partial mediation effect. Second, functionality also indirectly impacts creative satisfaction through creative autonomy, showing a significant mediation effect. Third, human-computer collaboration indirectly affects creative satisfaction through creative efficiency, also demonstrating partial mediation. Finally, human-computer collaboration indirectly affects creative satisfaction through creative autonomy, forming another significant mediation path. These results suggest that the functionality of AI tools and human-computer collaboration not only directly influence creative satisfaction but also play a key mediating role by improving creative efficiency and autonomy. Third, age, frequency of using generative video tools, and acceptance of new technology significantly impact creative satisfaction, while education level and frequency of using generative video tools have a significant effect on creative autonomy. Age and new technology acceptance also significantly affect creative efficiency.

Although this study reveals the impact of AI tool functionality, human-computer collaboration, and creative autonomy on filmmakers’ creative satisfaction and proposes multiple mediation paths, it still has some limitations that warrant further exploration.

First, the study primarily focuses on film creators, particularly directors, screenwriters, and editors, which limits the representativeness of the sample. While these creators have high levels of professionalism and experience in using AI tools, the study does not include non-professional creators or those from other creative fields, such as music and visual arts. Therefore, the external validity of the results needs further verification. Future research could expand to other creative fields to test the generalizability of AI tool functionality’s impact on creative satisfaction.

Second, while the survey method used in this study effectively collects large-scale data, as a self-reported data collection method, it may be influenced by individual biases. Specifically, creators’ self-assessments may differ from the actual creative process. Future studies could combine experimental research or real creative case analyses to improve the objectivity and accuracy of the research. Besides, it relies on cross-sectional survey data, which makes it difficult to establish strong causal relationships among the variables. The data were collected through self-reported questionnaires and may therefore be subject to social desirability bias. Third, the sample may be concentrated in specific regions or online platforms, which could limit the generalizability of the findings. This study does not distinguish among different types of artificial intelligence tools, and potential differences in their functions and usage contexts were not taken into account.

Additionally, the AVE values for Human-AI Collaboration (0.489) and Learning Cost (0.467) are slightly below the conventional 0.50 threshold, which is acknowledged as a limitation. Future research could refine these measurement items. Furthermore, the cross-sectional design limits the ability to establish strict causal ordering. The proposed directionality should be further tested using longitudinal or experimental designs.

Third, although this study explores gender differences in the preferences of male and female directors when using AI tools, the gender analysis is limited to simple categorical comparisons and does not delve into the deeper mechanisms of how gender influences the use of AI tools in the creative process. Future research could further investigate how gender, through various factors such as social-cultural influences and gender identity, impacts creative satisfaction and the effectiveness of AI tools. Futhermore, with regard to sample structure, female respondents accounted for a relatively high proportion of the sample. This characteristic may be jointly related to the platform-based sampling method, the voluntary participation mechanism, and the data-screening procedure. In addition, some male respondents were excluded during the data-cleaning process because they did not pass the validity checks or did not meet the criteria for the target population, which further affected the final gender distribution of the sample to some extent. Although the main purpose of this study is to examine the relationships among AI tool attributes, creative efficiency, creative autonomy, and creative satisfaction, rather than to provide a descriptive estimate of the overall population structure of the film industry, the gender composition of the sample may still deviate from the actual industry distribution. Therefore, caution is needed when generalizing the findings more broadly. Future research could improve the external validity and generalizability of the findings by adopting a more balanced sample structure or a more representative sampling strategy.

Fourth, this study focuses on the immediate effects of AI tools during the creative process but does not explore the long-term impact of AI tools on creators’ attitudes and creative abilities. AI tools may have a profound impact on creators’ long-term creative habits, innovation capacity, and working methods, which needs to be validated through longitudinal studies. Therefore, future research could explore the long-term effects of AI tools on creators’ creative behavior and cognitive patterns to address the gaps in the current study.

Furthermore, in terms of sample definition, the study treats directors, screenwriters, editors, and visual effects artists as a broader group of film creators. Although these professions all belong to the core creative workforce in filmmaking and share common experiences related to the integration of AI tools into creative practice, they may still differ substantially in their task structures, creative processes, and patterns of AI use. As a result, treating them as a relatively homogeneous group may reduce the explanatory precision of the findings with regard to occupation-specific differences. Future research could address this issue by including occupational type as a control variable or by conducting subgroup analyses to examine whether the mechanisms identified in this study vary across different professional roles within filmmaking.

Lastly, different types of filmmaking may differ in task structure, production process, and functional demands for artificial intelligence tools. However, this study does not further distinguish among different types of filmmaking, nor does it identify in detail the specific creative tasks for which respondents found artificial intelligence to be most helpful. Therefore, the present findings mainly reflect a general analysis of filmmakers’ overall experience with artificial intelligence use, rather than fully revealing the differentiated role of artificial intelligence across different creative tasks and filmmaking types. Future research may combine qualitative interviews or case studies and work with filmmaking experts to explore the specific functional value of artificial intelligence at different stages of the creative process, thereby enriching the findings of this study in a more detailed way.

## Data Availability

The original contributions presented in the study are included in the article/supplementary material, further inquiries can be directed to the corresponding author.

## References

[ref1] Alshamy A. Al-Harthi A. S. A. Abdullah S. (2025). Perceptions of generative AI tools in higher education: insights from students and academics at Sultan Qaboos University. Educ. Sci. 15:501. doi: 10.3390/educsci15040501

[ref2] Amabile T. M. (1996). Creativity in Context: Update to the Social Psychology of Creativity. Boulder: Westview Press.

[ref3] Bandura A. (1997). Self-Efficacy: The Exercise of Control. New York: W. H. Freeman.

[ref6] Cox S. R. Djernæs H. B. van Berkel N. (2025). Beyond productivity: rethinking the impact of creativity support tools. arXiv. doi: 10.48550/arXiv.2505.01601

[ref5] Csikszentmihalyi M. (1990). Flow: The Psychology of Optimal Experience. New York: Harper & Row.

[ref7] Davis F. D. (1989). Perceived usefulness, perceived ease of use, and user acceptance of information technology. MIS Q. 13, 319–340. doi: 10.2307/249008

[ref8] Deci E. L. Ryan R. M. (2000). The “what” and “why” of goal pursuits: human needs and the self-determination of behavior. Psychol. Inq. 11, 227–268. doi: 10.1207/S15327965PLI1104_01

[ref9] Doshi A. R. Hauser O. P. (2024). Generative AI enhances individual creativity but reduces diversity of ideas. Sci. Adv. 10:eadn5290. doi: 10.1126/sciadv.adn5290 38996021 PMC11244532

[ref10] Engelbart D. C. (1962). Augmenting Human Intellect: A Conceptual Framework (Summary Report AFOSR-3223). Menlo Park: Stanford Research Institute.

[ref9001] Fornell C. Larcker D. F. (1981). Evaluating structural equation models with unobservable variables and measurement error. Journal of marketing research, 18, 39–50.

[ref11] Goodhue D. L. Thompson R. L. (1995). Task-technology fit and individual performance. Inf. Syst. Res. 6, 293–312. doi: 10.1287/isre.6.4.293

[ref12] Heigl R. (2025). Generative artificial intelligence in creative contexts: a systematic review and future research agenda. Manag. Rev. Q. 76, 955–992. doi: 10.1007/s11301-025-00494-9

[ref9002] Henseler J. Ringle C. M. Sarstedt M. (2015). A new criterion for assessing discriminant validity in variance-based structural equation modeling. Journal of the academy of marketing science, 43, 115–135.

[ref13] Hosanagar K. Ahn D. (2024). Designing human and generative AI collaboration. arXiv:2412.14199 (Preprint).

[ref14] Jiang T. (2024). Human-AI interaction research agenda: a user-centered perspective. Comput. Educ.: Artif. Intell. 5:100123. doi: 10.1016/j.caeai.2024.100123

[ref9003] Kock N. (2015). Common method bias in PLS-SEM: A full collinearity assessment approach. International Journal of e-Collaboration (ijec), 11, 1–10.

[ref15] Licklider J. C. R. (1960). Man-computer symbiosis. IRE Trans. Hum. Factors Electron. HFE-1, 4–11. doi: 10.1109/THFE2.1960.4503259

[ref16] Lin Z. Ng Y. L. (2024). Unraveling gratifications, concerns, and acceptance of creative satisfaction in AI technology adoption. Int. J. Hum. -Comput. Interact. 41, 10725–10742. doi: 10.1080/10447318.2024.2436749

[ref17] Maier S. Schneider M. Feuerriegel S. (2025). Partnering with generative AI: experimental evaluation of human-led and model-led interaction in human-AI co-creation. arXiv. doi: 10.48550/arXiv.2510.23324

[ref18] McGuire J. De Cremer D. Van de Cruys T. (2024). Establishing the importance of co-creation and self-efficacy in creative collaboration with artificial intelligence. Sci. Rep. 14:18525. doi: 10.1038/s41598-024-69423-2, 39122865 PMC11316096

[ref9004] Podsakoff P. M. MacKenzie S. B. Lee J. Y. Podsakoff N. P. (2003). Common method biases in behavioral research: a critical review of the literature and recommended remedies. Journal of applied psychology, 88:879. 14516251 10.1037/0021-9010.88.5.879

[ref19] Rafner J. Zana B. Bang Hansen I. Ceh S. Sherson J. Benedek M. (2025). Agency in Human-AI Collaboration for Image Generation and Creative Writing: Preliminary Insights from Think-Aloud Protocols. Creativity Research Journal, 1–24.

[ref20] Rajcic N. (2024). “Towards a diffractive analysis of prompt-based generative AI interfaces,” in Proceedings of the ACM Conference on Human Factors in Computing Systems.

[ref21] Rezwana J. Ford C. (2025). Improving user experience with FAICO: towards a framework for AI communication in human-AI co-creativity. arXiv:2504.02526. doi: 10.48550/arXiv.2504.02526

[ref22] Singh S. Hindriks K. Heylen D. Baraka K. (2025). A systematic review of human-AI co-creativity. arXiv:2506.21333. doi: 10.48550/arXiv.2506.21333

[ref23] Soylu M. Y. Lee J. Hung J.-T. Zhang C. C. Joyner D. A. (2025). AI literacy as a key driver of user experience in AI-powered assessment: insights from Socratic mind. arXiv. doi: 10.48550/arXiv.2507.21654

[ref24] Tang Y. Zhang N. Ciancia M. Wang Z. (2024). “Exploring the impact of AI-generated image tools on professional and non-professional users in the art and design fields.,” in In Companion Publication of the 2024 Conference on Computer-Supported Cooperative Work and Social Computing (pp. 451–458)

[ref26] Wang N. Kim H. Peng J. Wang J. (2025). Exploring creativity in human–AI co-creation: a comparative study across design experience. Front. Comput. Sci. 7:1672735. doi: 10.3389/fcomp.2025.1672735

[ref27] Wang P. Khinvasara Y. Creijghton G. J. Scholing T. Wang Y. Zhou Z. . (2025). Enhancing designer creativity through human–AI co-ideation: a co-creation framework for design ideation with custom GPT. AI EDAM 39:e22. doi: 10.1017/S0890060425100127, 41292463

[ref28] Wang W.-F. Lu C.-T. Campanyà N. P. Chen B.-Y. Chen M. Y. (2025). “AIdeation: designing a human-AI collaborative ideation system for concept designers,” in Proceedings of the ACM Conference on Computer-Supported Cooperative Work and Social Computing.

